# Redesigning Vina@QNLM for Ultra-Large-Scale Molecular Docking and Screening on a Sunway Supercomputer

**DOI:** 10.3389/fchem.2021.750325

**Published:** 2021-10-28

**Authors:** Hao Lu, Zhiqiang Wei, Cunji Wang, Jingjing Guo, Yuandong Zhou, Zhuoya Wang, Hao Liu

**Affiliations:** ^1^ College of Computer Science and Technology, Ocean University of China, Qingdao, China; ^2^ Pilot National Laboratory for Marine Science and Technology, Qingdao, China

**Keywords:** molecular docking, virtual screening, parallel computing, Sunway supercomputer, drug discovery

## Abstract

Ultra-large-scale molecular docking can improve the accuracy of lead compounds in drug discovery. In this study, we developed a molecular docking piece of software, Vina@QNLM, which can use more than 4,80,000 parallel processes to search for potential lead compounds from hundreds of millions of compounds. We proposed a task scheduling mechanism for large-scale parallelism based on Vinardo and Sunway supercomputer architecture. Then, we readopted the core docking algorithm to incorporate the full advantage of the heterogeneous multicore processor architecture in intensive computing. We successfully expanded it to 10, 465, 065 cores (1,61,001 management process elements and 0, 465, 065 computing process elements), with a strong scalability of 55.92%. To the best of our knowledge, this is the first time that 10 million cores are used for molecular docking on Sunway. The introduction of the heterogeneous multicore processor architecture achieved the best speedup, which is 11x more than that of the management process element of Sunway. The performance of Vina@QNLM was comprehensively evaluated using the CASF-2013 and CASF-2016 protein–ligand benchmarks, and the screening power was the highest out of the 27 pieces of software tested in the CASF-2013 benchmark. In some existing applications, we used Vina@QNLM to dock more than 10 million molecules to nine rigid proteins related to SARS-CoV-2 within 8.5 h on 10 million cores. We also developed a platform for the general public to use the software.

## Introduction

The virtual screening technology can be employed to obtain lead compounds from a large number of candidate compounds, thus significantly reducing the cost of drug discovery (Pagadala et al., 2017). Currently, the molecular docking technology is one of the most commonly used virtual screening technology. The accuracy of virtual screening depends on the scale of molecular docking. The mainstream ZINC15 database in the field of drug screening currently contains 750 million compounds that can be purchased (Irwin et al., 2012). To determine the ideal lead compounds in molecular docking, each target needs to be docked with each compound. In addition, more than 20,000 drug targets have been identified. In such a massive computing scenario, if only a single computer or small cluster is used for docking, then the running computing time is unacceptable. Therefore, large-scale parallel computing is required to solve this problem.

Super-large-scale molecular docking parallel computing has large bottlenecks in multiple links, such as input/output (I/O), task allocation, and communication. To realize large-scale parallel computing, we need to encapsulate a series of interfaces at the job scheduling level. Meanwhile, the massive read and write processes overwhelm the file system, and the necessary integration and optimization of I/O operations must be conducted. The thread and cache utilization of the docking algorithm should be optimized to totally improve parallelism while reducing inefficient operations, such as access and storage (Dong et al., 2018). The accuracy of virtual screening, such as molecular docking, is highly dependent on the size of the candidate molecular library and the performance of the screening algorithm. The misprediction of early lead compounds can be costly and time-consuming in the subsequent stages of drug development. The molecular docking algorithm estimates the most likely binding structure and energy between a pair of ligands and receptors. Currently, we aim to obtain the ideal conformation by considering the atomic space interaction, hydrophobic interaction, hydrogen bonding, and other factors. However, excessive physical descriptors limit the running speed, thus affecting the screening scale and reducing the screening accuracy. Therefore, it is necessary to reasonably design a docking algorithm and improve the speed of molecular docking and the screening scale (Zhang et al., 2013; Li et al., 2019).

The first molecular docking software DOCK (Allen et al., 2015) was developed in 1980. However, because of the limitations of the algorithm in this software, its calculation accuracy is poor. In addition, it is a serial program with limited computing power. Accordingly, a parallel strategy was introduced to solve the computing efficiency problem. The message passing interface (MPI) was introduced to achieve process-level parallelism using a large number of cores to reduce the docking completion time (e.g., VinaMPI) (Nocedal and Wright, 1999; Ellingson et al., 2013; Liu et al., 2020; Zhang et al., 2013). At the same time, the docking efficiency is improved through thread-level parallelism. Multiple CPUs of a single processor—each of which simultaneously performs optimized search algorithms—implement thread-level parallelism [e.g., AutoDock Vina (herein referred to as Vina)] (Trott and Olson, 2009).

In the past decade, graphical processing units (GPUs) have shown superior performance in the field of image processing. In the field of molecular docking, a GPU is gradually introduced to facilitate the computation. For example, MolDock (Thomsen and Christensen, 2006) was used in the multithreading technology to accelerate the calculation process. Heterogeneous multicore systems adopt the main processor + coprocessor architecture. In this architecture, the main processor handles complex logic control tasks, such as the distribution of molecular docking tasks and reading of files. The coprocessor processes the computational part of the molecular docking and docking algorithm that performs multiple iterations. This type of computation has a high density and a simple logical branch of large-scale data. The two works together provide an efficient computing platform for specific applications, and different parallelism strategies can be used to solve different problems in the field of molecular docking (Altuntaş et al., 2016). Heterogeneous multicore architectures have advantages in the field of molecular docking owing to their unique architecture (Fu et al., 2016; Chen et al., 2017; Xiao et al., 2021).

In this study, we successfully extended the molecular docking system to 10 million cores in Sunway supercomputer by introducing new strategies and algorithms and demonstrate parallelism in the molecular docking. We evaluated our algorithms and strategies on Sunway supercomputer, a heterogeneous multicore processor system. Experimental results show that the docking accuracy is better than that of traditional molecular docking methods and has a good parallelism efficiency and computation speed. Our main contributions are as follows:

• We added an ultra-large-scale molecular docking scheduling parallelism for large-scale parallelism based on Vinardo’s and Sunway supercomputer architecture. We managed to scale to 1,61,001 management processing elements (MPEs) and 10,304,064 computing processing elements (CPEs) with a strong scalability of 55.92%.
• We readopted the core docking algorithm to take full advantage of the heterogeneous multicore processor architecture in intensive computing. The introduction of the heterogeneous multicore processor architecture achieved the best speedup, which is 11x more than that of the management process element of Sunway. The accuracy of the software was evaluated using the CASF-2013 and CASF-2016 benchmarks.
• We developed a docking platform, including core docking functions, to meet the docking needs of general users. Existing docking practices include docking more than 10 million molecules to nine rigid proteins about SARS-CoV-2 within 8.5 h.

## Background

### Sunway’s Processor

Sunway’s processor was developed based on the previous-generation SW26010 processor. Sunway’s multicore processors integrate several core groups (CGs), each of which contains an MPE and an array of 8 × 8 CPEs, which are connected through a ring network. MPEs mainly include computation, control, communication, and I/O functions. CPEs are mainly used for computation. A CPE and MPE are called a master core and slave core, respectively. The CPEs in the CGs are interconnected with one another and with external interactions through an intra-array network. Data communication (peer-to-peer and aggregate communication) between any two CPEs in the array can be performed via a register-level data communication method (see Supplementary Figure S1B).

The MPE uses an autonomous SW64 instruction set with a data cache. The computing processing element supports double-precision, single-precision, and half-precision floating-point computation and integer operations. Each CPE has its own independent instruction cache and data storage space. The data storage space can be configured as a fully user-controlled local data memory (LDM), or part of the data storage space can be configured as a hardware piece’s automatically managed local data cache (LDcache). The data transfer between the LDM and main memory can be achieved using the direct memory access (DMA) or conventional load/store instructions between the LDcache and main memory.

### Sunway’s Supercomputer

Sunway supercomputer system inherits and develops the architecture of “Sunway TaihuLight” based on high-performance heterogeneous multicore processors and interconnection network chips. The system consists of a computing system, interconnection network system, software system, peripheral service system, maintenance and diagnosis system, power supply system, and cooling system (see Supplementary Figure S1B).

The software system supports the management and scheduling of super-large-scale programming resources with more than 10 million cores; it provides system-wide monitoring management, fault-tolerant mechanisms, multilevel debugging, and performance tuning of application software. In addition, it supports parallel programming environments, such as MPI, OpenMP, and OpenAcc. Thus, it can boost the implementation of the molecular docking parallel strategy.

### Vina@QNLM

Vina is a popular molecular docking program in drug discovery at early stages and opts for the iterated local search global optimizer to search for the minimum affinity for molecular docking. Specifically, the software uses the Broyden–Fletcher–Goldfarb–Shanno (BFGS) method (Nocedal and Wright, 1999) to find the local optimum.

The value of the scoring function and its gradient, such as the derivative of the scoring function with respect to its parameters, are used in the BFGS algorithm. The parameters of the scoring function include the orientation and position of the small molecule and the values of the torsions for the active rotatable bonds. As for the binding energy, the sum of distance-dependent atom pair interactions is considered in Eq. 1:

$$E=\sum e_{pair}\left(sd\right).$$ (1)

Here, sd is the surface distance, which is equal to the difference between the interatomic distance and the radii of the atoms in the pair. There are five terms in total.

As can be seen in Eq. 2, the final predicted binding affinity is that each term is multiplied by a constant and then added to the sum. For more details, please refer to the original article by Trott and Olson (2009).

$$e_{\mathrm{pair}}\left(\mathrm{sd}\right)=w_{1}*\mathrm{Gaus}s_{1}\left(\mathrm{sd}\right)+w_{2}*\mathrm{Gaus}s_{2}\left(\mathrm{sd}\right)+w_{3}*\mathrm{Repulsion}\left(\mathrm{sd}\right)+w_{4}*\mathrm{Hydiophbic}+w_{5}*\mathrm{HBond}\left(\mathrm{sd}\right).$$ (2)

Vinardo (Quiroga and Villarreal, 2016) is based on AutoDock Vina and uses a simpler scoring function, while improving the docking, scoring, ranking, and virtual screening results of the scoring function. First, the main difference of Vinardo is that the second minimum, which is physically unreasonable in the Vina space interaction, is eliminated, and then, Vinardo adjusts atomic radii, such as nitrogen and oxygen aliphatic carbons, to further improve the performance of the scoring function. Vinardo uses a combination of Gaussian gravity and quadratic repulsion to manage steric interactions. Finally, a hydrophobic term in Vinardo, which is approximately the same as the diameter of a water molecule, can be obtained. The scoring function of Vinardo shows good performance on the CASF-2013 benchmark (Li et al., 2018).

In our implementation, we present a molecular docking piece of software called Vina@QNLN, which adopts the scoring function of Vinardo and is reconstructed based on the architectures of Vina and Sunway supercomputer. We added an ultra-large-scale molecular docking scheduling parallelism.

## Design and Optimization

In this section, we introduce the parallel implementation of Vina@QNLM on Sunway supercomputer in detail. A two-level parallelization strategy, including the combination of the master–master core and multi-thread heterogeneous parallelism of a new processor on Sunway supercomputer, is introduced to achieve the scalability and speedup of Vina@QNLM.

### Large-Scale Parallelism for Molecular Docking Tasks

In the process of ultra-large-scale docking, scheduling and I/O operations have been a problem. First, this is a typical many-to-many problem. Multiple targets are required, and there are multiple pocket files. Each molecule must be docked with the target. Second, the program needs to be carefully designed so that pocket information and grid data can be reused. To reduce the number of I/O operations, the data must be packaged and stored.

There are a lot of previous successful experiences in parallel computing on Sunway [e.g., Parallel CFD Simulation Software, reactive force field simulation, and parallel implementation of the discontinuous Galerkin density functional theory (DGDFT) on Sunway’s supercomputer] (Tsutsui and Collet, 2013; Gao et al., 2020; Hu et al., 2021). According to the current software foundation, we employed MPI for data transmission, which supports point-to-point communication and is mature on Sunway supercomputer.

**FIGURE 1:**
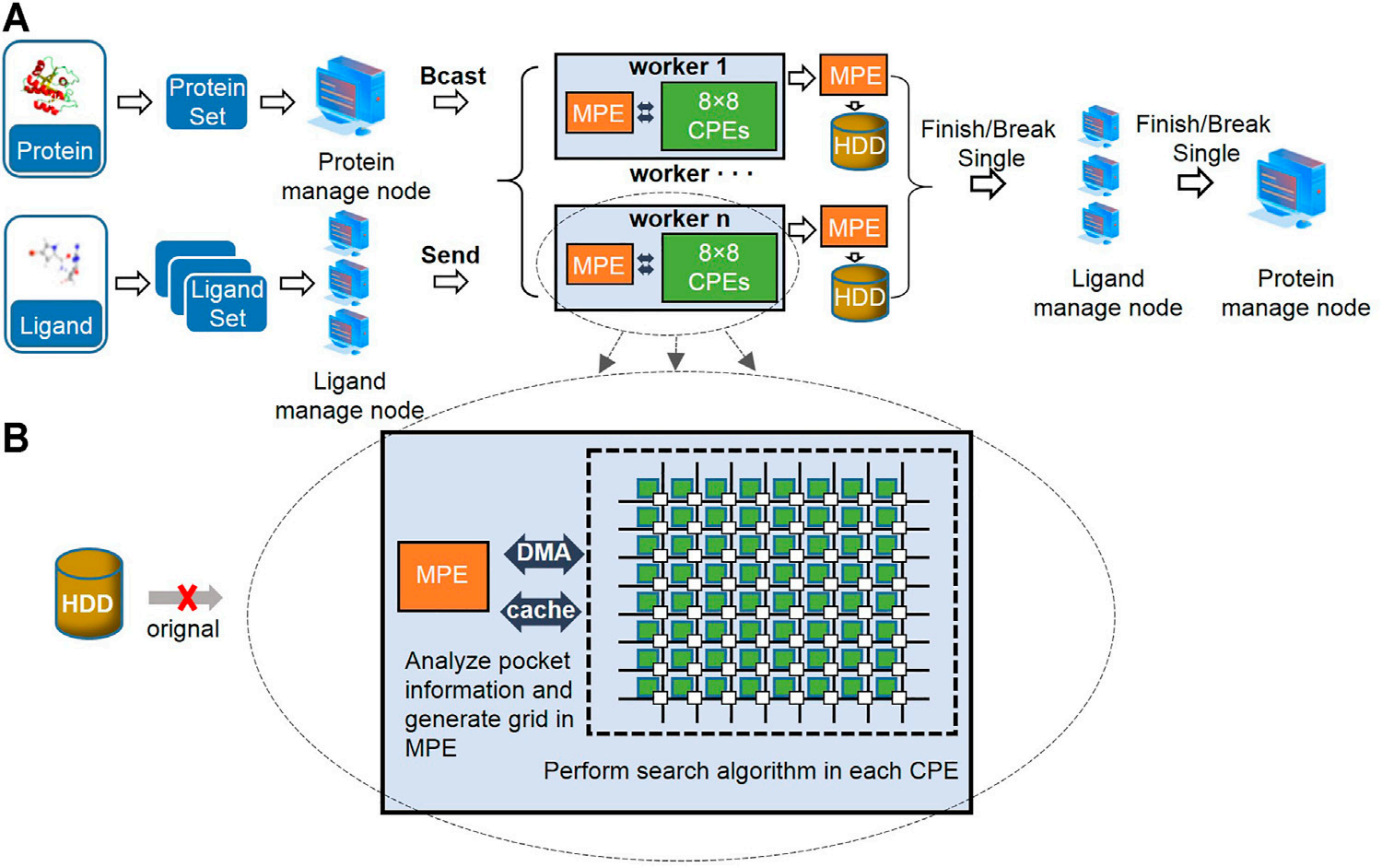
Parallel framework of Vina@QNLM based on Sunway supercomputer, large-scale parallelism for molecular docking tasks **(A)**, and data communication and optimization between MPE and CPE **(B)**.

As the first level of parallelism, we adopted MPI to realize the docking of proteins and ligands between different MPEs, addressing the task scheduling and allocation problems (see Figure 1A). First, a process called the “protein manage node” was allocated to read all the target and pocket information in batches, and subsequently, the information was broadcasted to each work node. Thereafter, a certain amount of “ligand manage node” was allocated to read small-molecule information, thus realizing the reuse of pocket and grid information. The number of ligand manage nodes must be carefully selected. If it is considerably large, then it will cause a decrease in computing efficiency; otherwise, it will cause a performance bottleneck. The size of the PDBQT format data of small molecules is approximately 1,500 bytes, and the number of ligand manage nodes is positively correlated with the total number of processes. Thus, we selected the total number of processes/ligand manage nodes as ∼300. Subsequently, a certain number of small molecules were evenly distributed to each work node to form a small-molecule library. The number of molecules that each ligand manages to send to the worker was calculated by Eq. 3, where nl, ntp, and nlm represent the number of molecules in each ligand set, the number of all processes, and the number of ligand manage nodes, respectively. By docking each target with the molecules in the small-molecule library, docking simulation data were generated. Thereafter, the results were returned to the MPE, and the docking result data were saved. The MPE in the worker sends a finish or break signal to the ligand manage node, and the ligand manage node sends the signal to the protein manage node. Finally, the program ends.

$$f\left(x\right)=\frac{n_{l}}{\left(d_{\mathrm{lp}}-1-n_{\mathrm{lm}}\right)*n_{\mathrm{lm}}\;}.$$ (3)

To ensure that each process remains busy and reduces the idle time based on the characteristics of multicores and heterogeneity of the processor and to increase the I/O efficiency, a multilevel processing scheme was adopted to separately open up multiple processes to read a large number of small-molecule input data files, target data files, and pocket files. This process plays the role of a scheduler. Because the target data are cumbersome and have many coordinates, when a large number of targets need to be docked, it may result in an insufficient stack space. Therefore, we gradually eliminated the target data that had completed docking, thereby reducing the number of times the file was read, while increasing the I/O efficiency.

### Adaptation of the Molecular Docking Algorithm

The original strategy is that each MPE uses a hard disk to read the data. However, this approach causes performance bottlenecks. Through the scheduling strategy mentioned in Section 3.1, the target data can be reused considerably. Here, the MPE in a CG reads pocket information in the memory and generates grid data. This information can be reused for the same protein. Considering the docking time and efficiency, we transplanted the most time-consuming search algorithms as a second level of parallel and adopted the Monte Carlo simulated annealing algorithm using time as the random number seed to search for the conformation of the molecule. The algorithm also maintains a high computational efficiency, accuracy of the docking results, and speed of large-scale docking. Inspired by Vina, we executed algorithms on each CME (Figure 1B). We employed the BFGS algorithm to determine the minimum local energy for the optimal local spatial orientation and location information. This process is accompanied by data interactions by the DMA and cache method. It reserves the best 20 poses in each CME and then transfers them to the MPE. After comparison, the MPE retained the specified poses.

### Acceleration of the Docking Algorithm Based on Sunway

The CPE contains local data management (LDM). To achieve a high memory bandwidth, the CPE core accesses the data by directly accessing the main memory or transferring the data to the LDM through a DMA operation and then loads it from the LDM. Three types of data should be loaded into the LDM: configuration information, parameters, and molecular coordinates. These configurations were loaded into the LDM prior to the task cycle. Therefore, during the actual conformation search and position-matching process, the data could be accessed quickly.

The values of the four parameters, including the van der Waals force and Gauss term between the receptor and ligand atoms, are not related to the small ligand molecule. The software distributes the four parameters in a virtual three-dimensional grid containing a receptor small molecule. The data volume of the energy grid is too large to be completely incorporated into the local memory of the CPE. For the conformation search and energy calculation process, we used the cache method to access the main memory data to facilitate the docking calculation. The force, which is a combination of quadratic repulsion and Gaussian gravity to handle steric interactions, adopted the same method.

## Results and Discussion

### Scoring Function and Sample Power

To evaluate the accuracy of the scoring function, we conducted evaluations on the CASF-2013 (Li et al., 2018) and CASF-2016 benchmarks (Su et al., 2019). The two benchmarks evaluate scoring functions using four metrics: ranking power, scoring power, docking power, and screening power. We also added another metric: sample power for evaluating Vina@QNLM predicting the ligand pose. In this article, we only briefly review the definitions; a more precise and complete definition can be found in the CASF-2013 article (Li et al., 2014) and CASF-2016 article (Su et al., 2019).

#### Evaluation on the CASF-2013 Benchmark

The CASF-2013 benchmark consists of 195 ligand–receptor complex structures with associated affinities. In addition, 16,123 computer-generated ligand poses were provided for docking power evaluation and 6,36,010 for screening power evaluation. The correctness of Vina@QNLM after transplantation was verified using the entire CASF-2013 benchmark, and it was added to 27 docking software models with the results of the CASF-2013 test for a comparative analysis.

##### Scoring Power and Ranking Power

**FIGURE 2:**
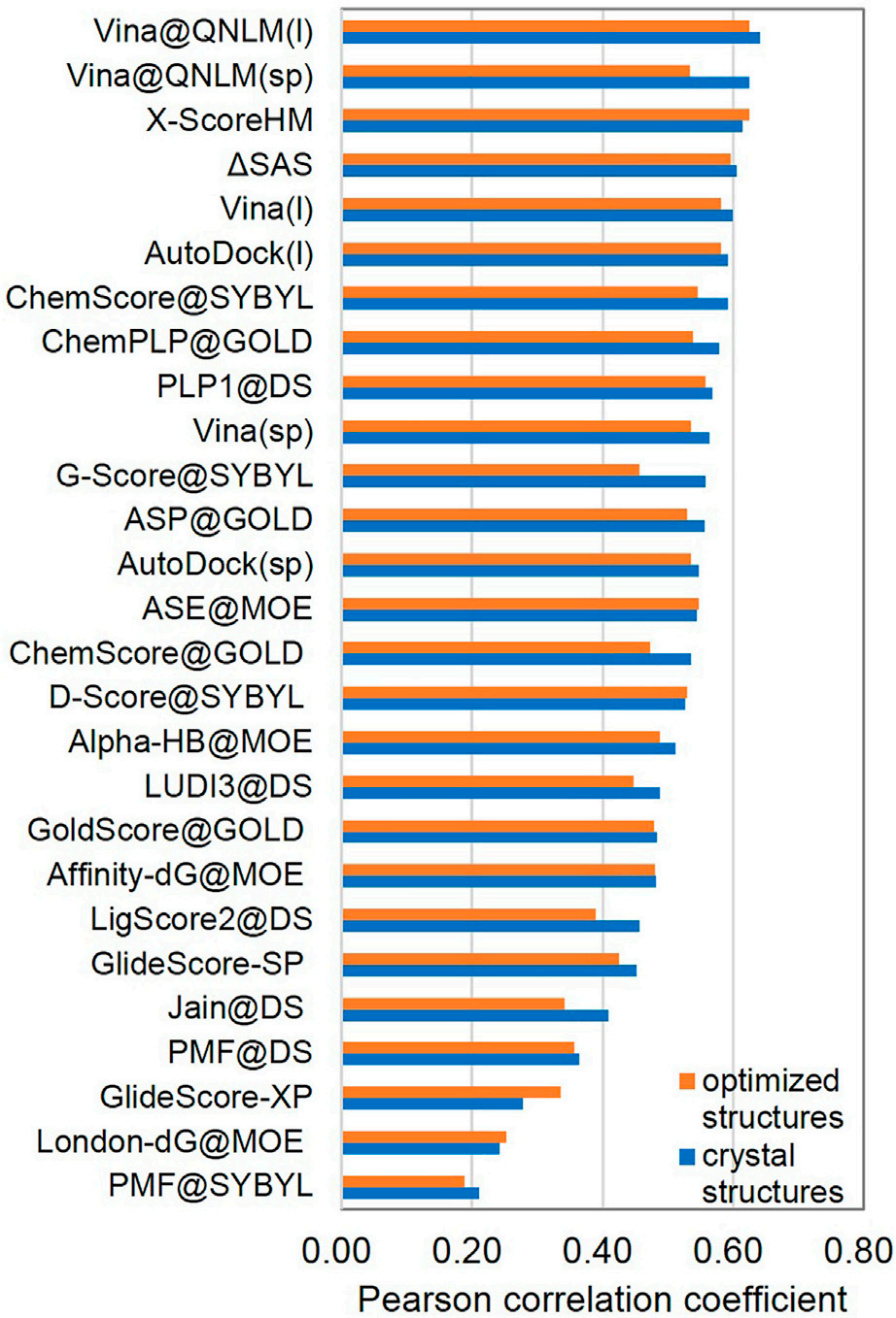
Scoring power of crystal structures and optimized structures and Pearson correlation coefficients of Vina@QNLM in single-point (sp) and local (l) modes (blue and orange bars), compared to the methods evaluated in CASF-2013 and recalculated.

**FIGURE 3:**
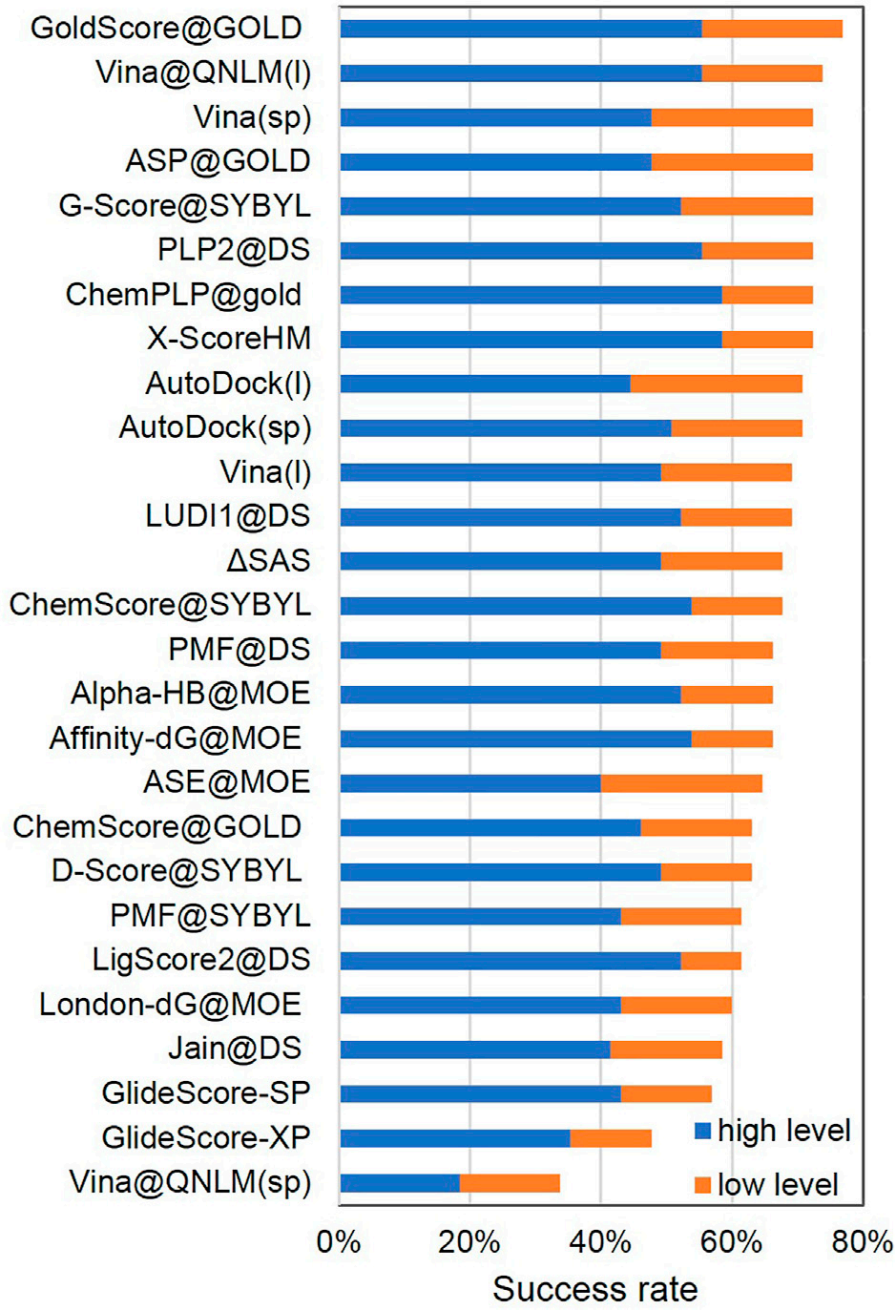
Ranking power of high- and low-level success rate of Vina@QNLM in single-point (sp) and local (l) modes (blue and orange bars), compared to the methods evaluated in CASF-2013 and recalculated.

The scoring power (see Figure 2; Supplementary Table S1) determines whether a score generated by the scoring function in Vina@QNLM could be linearly correlated with the experimental binding constants. Pearson’s correlation coefficient was used to measure this metric. The ranking power (see Figure 3; Supplementary Table S2) evaluates the ability of a scoring function to correctly rank the ligands of a target protein based on their experimental affinities. The ranking power is measured by the “high-level” success rate, and the number of targets where the three complexes are correctly ranked is counted (i.e., best > median > poorest). A “low-level” success rate is counted with a relaxed criterion (best > median and best > poorest). For the scoring power in single-point and local modes, Vina@QNLM achieved correlation coefficients of 0.625 and 0.641 for crystal structures and 0.534 and 0.624 for optimized structures. Compared to the 27 methods, Vina@QNLM is the best in relation to experimental data. In addition, the two modes of Vina@QNLM showed two extreme results in terms of the ranking power. The local mode showed the best success rate in the high and low levels, whereas the single-point mode showed the worst success rate.

##### Docking Power and Screening Power

**FIGURE 4:**
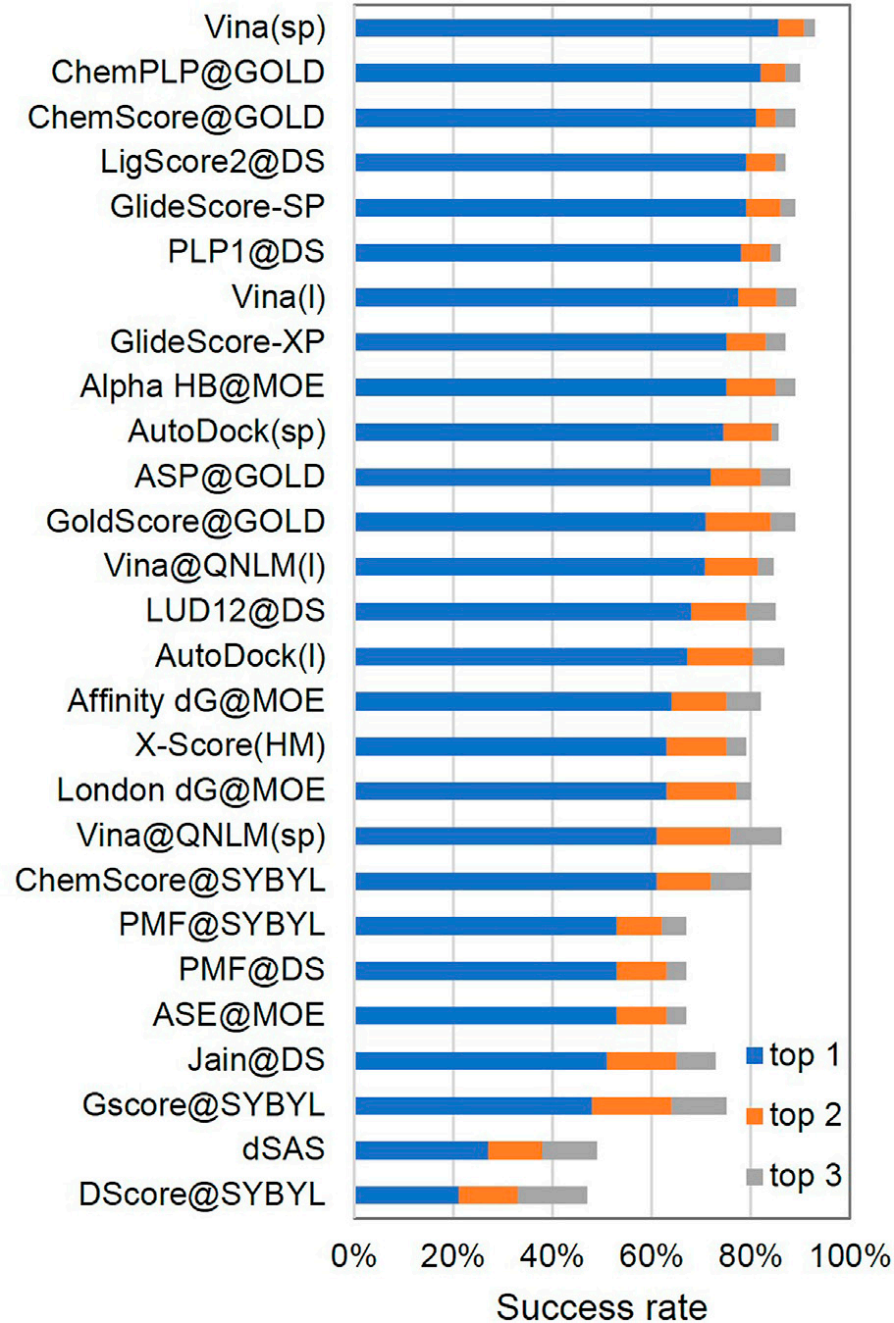
Docking power of top 1 (1%), top 2 (5%), and top 3 (10%) success rates of Vina@QNLM in single-point and local modes (blue, orange, and gray bars), compared to the methods evaluated in CASF-2013 and recalculated.

The docking power is used to evaluate the ability of Vina@QNLM to distinguish between the native pose and decoy ligand binding poses for a given receptor. If a ligand binding pose is less than or equal to 2 Å as compared to the experimental ligand pose, then we consider the pose to be native. Typically, a ligand pose is evaluated using the root-mean-square deviation (RMSD) and does not take into account the hydrogen atoms of the ligand. For each receptor, the poses considered include the computer-generated poses and ligand crystallographic structure. The docking power was measured by the success rate: the percentage of proteins with a native pose as the best (top 1), second (top 2), or third (top 3) scored pose. For the docking power, Vina@QNLM nearly ranked in the middle of the 27 methods (see Figure 4; Supplementary Table S3).

**FIGURE 5:**
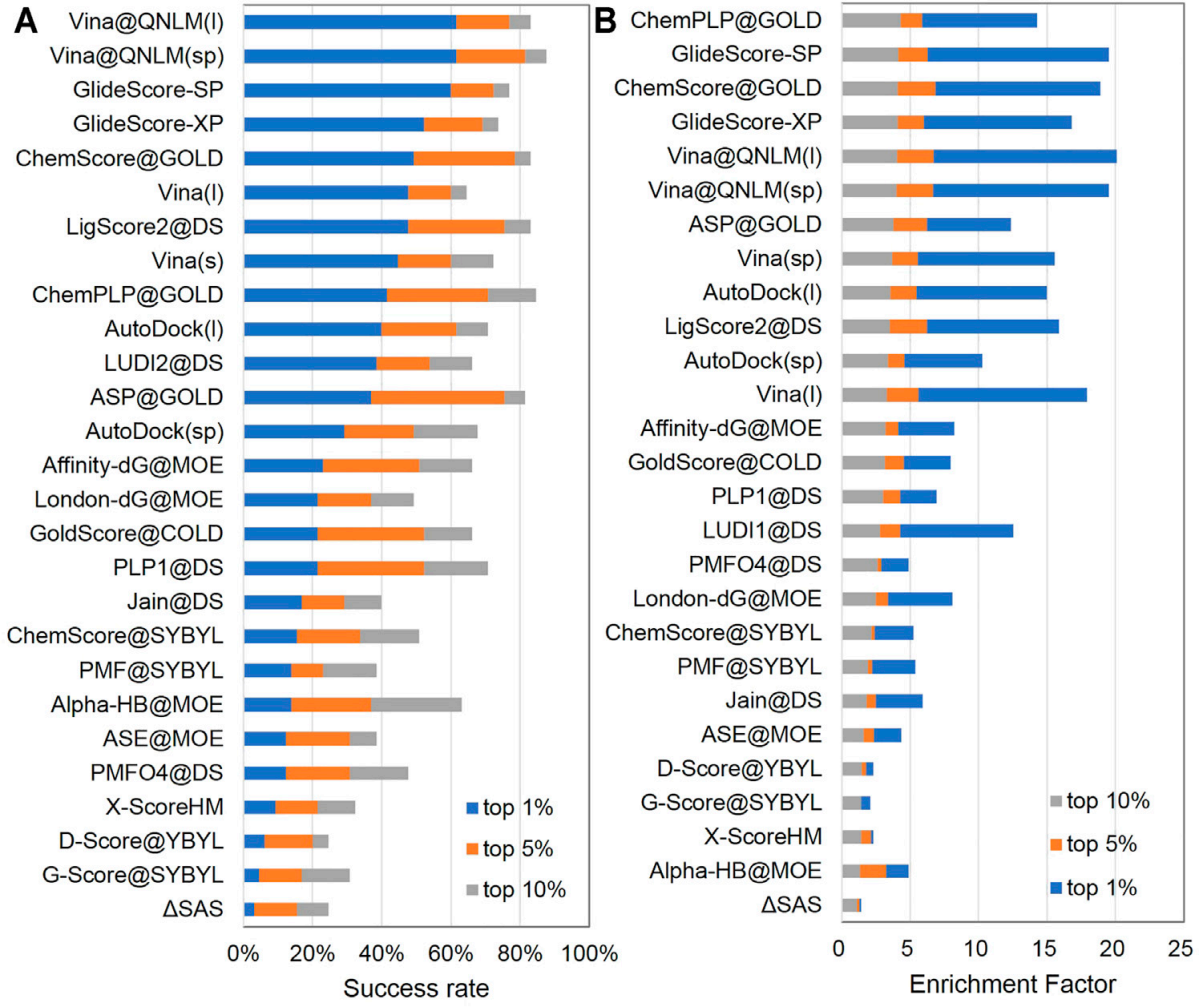
Screening power of top 1%, top 5%, and top 10% success rates **(A)** and enrichment factors **(B)** of Vina@QNLM in single-point (sp) and local (l) modes, compared to the methods evaluated in CASF-2013 and recalculated.

The purpose of the screening power is to evaluate the ability of the molecular docking software to separate the two ligands. We adopt the enrichment factor and success rate to assess the screening powder of our molecular docking software. The enrichment factor is defined as the number of binder ligands among the best 1% (top 1%), 5% (top 5%), or 10% (top 10%) scored poses, divided by the total number of binder ligands times 1, 5, or 10%. Among the 27 methods, Vina@QNLM was almost the best (see Figure 5A; Supplementary Table S4). The success rate is the percentage of targets for which the ligand with the best experimental affinity is among the best 1% (top 1%), 5% (top 5%), or 10% (top 10%) scored poses. Vina@QNLM is at the top among the 27 methods (see Figure 5B; Supplementary Table S5).

##### Sample Power

**FIGURE 6:**
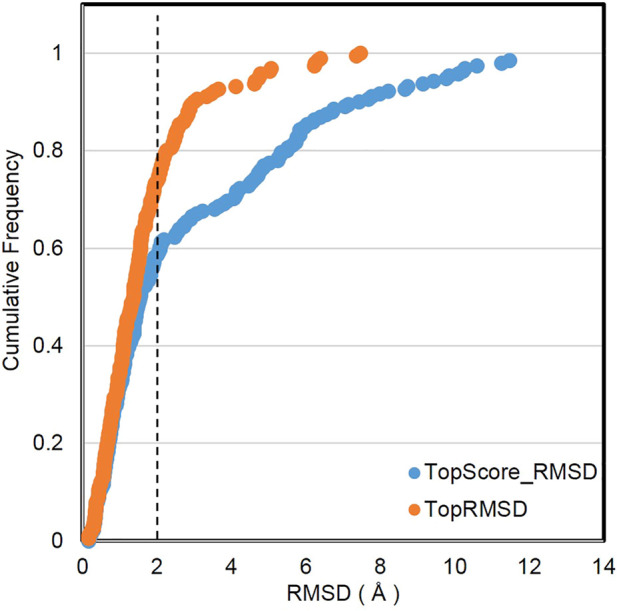
Sample power by comparing cumulative frequencies of TopScore_RMSD and TopRMSD, compared to the methods evaluated in CASF-2013 and recalculated.

The sample power refers to the ability of the molecular docking software to predict the binding poses of a ligand. We used RMSD to evaluate the sampling power. The prediction of Vina@QNLM is regarded to be successful if the RMSD between the native binding pose and docked binding pose is below 2.0 Å. Two methods were used to evaluate the sample capability in our work, including the highest conformation generated by the molecular docking software (referred to as the top-score RMSD) and the closest conformation to the native binding pose (referred to as the top RMSD). The figure indicates that the success rate of Vina@QNLM for the top-scored pose is 59%, but that for the top RMSD pose is significantly higher (73%) (see Figure 6). With reference to other molecular docking software (not the same benchmark) (see Wang et al., 2016), Vina@QNLM is in between the middle to upper levels.

#### Evaluation on the CASF-2016 Benchmark

We also use the updated benchmark to evaluate the scoring function of Vina@QNLM. CASF-2016 is basically the same as the CASF-2013 benchmark, only slightly different in some indicators. The same data were prepared with some definitions of terms that we do not describe here but can be referred to Wang et al. (2016).

##### Scoring Power and Ranking Power

**FIGURE 7:**
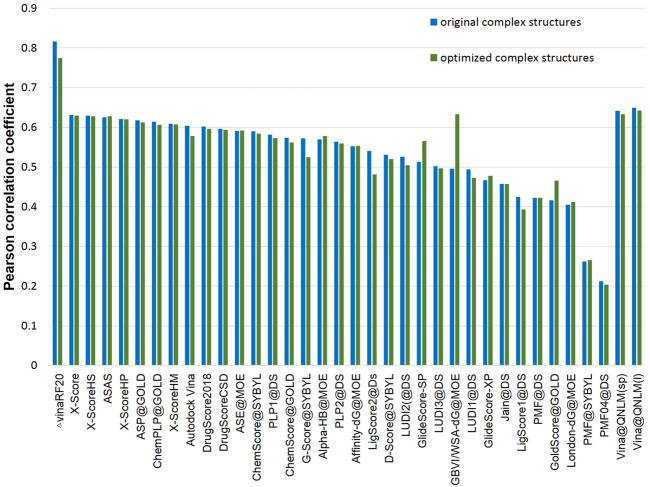
Scoring power of original complex structures and optimized complex structures and Pearson correlation coefficients of Vina@QNLM in single-point (sp) and local (l) modes (blue and green bars), compared to the methods evaluated in CASF-2016 and recalculated.

The scoring power of the scoring function was measured by the correlation between the calculated binding score and the experimental binding constants of a given protein ligand. Supplementary Table S6 shows the Pearson correlation coefficient and standard deviation (SD) generated by 35 scoring functions. Figure 7 shows the evaluation results of all scoring functions on the structure of the original crystal complex. The highest Pearson linear correlation coefficient is that of ΔVinaRF_20_, that is, 0.816, significantly higher than those of other scoring functions. The second highest is Vina@QNLM, whose Pearson linear correlation coefficients are 0.641 and 0.649 in the single-point and local modes, respectively.

In addition to using the original crystal complex structure, the scoring ability of the locally optimized complex structure was also tested. The Pearson correlation coefficients and SDs are presented in Supplementary Tables S6, S7; Figure 7. The Pearson correlation coefficient of ΔVinaRF_20_ is the highest among the 35 scoring functions, reaching 0.774, followed by Vina@QNLM, that is, 0.633 and 0.642 in the single-point and local modes, respectively.

**FIGURE 8:**
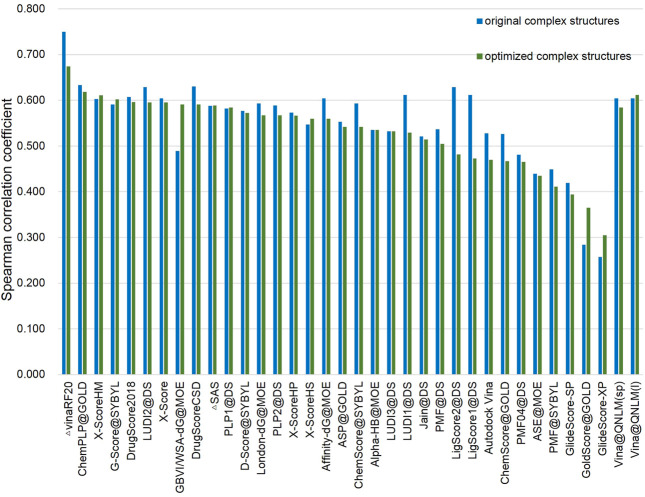
Scoring power of original complex structures and optimized complex structures and Spearman’s correlation coefficients of Vina@QNLM in single-point (sp) and local (l) modes (blue and green bars), compared to the methods evaluated in CASF-2016 and recalculated.

In the CASF-2016 benchmark, the average Spearman rank correlation coefficient (ρ), Kendall correlation coefficient (τ), and prediction index (PI) are equivalent for the score function. Accordingly, the Spearman rank correlation coefficient is shown in Figure 8; Supplementary Tables S8, S9. Vina@QNLM scoring function ranked fourth and fifth, and its Spearman's rank correlation coefficient was 0.616 and 0.604 in the single-point and local modes. After the local optimization of the compound structure, its Spearman's rank correlation coefficients were 0.584 and 0.612, respectively, ranking 10th and second in the single-point and local model.

##### Docking Power and Screening Power

**FIGURE 9:**
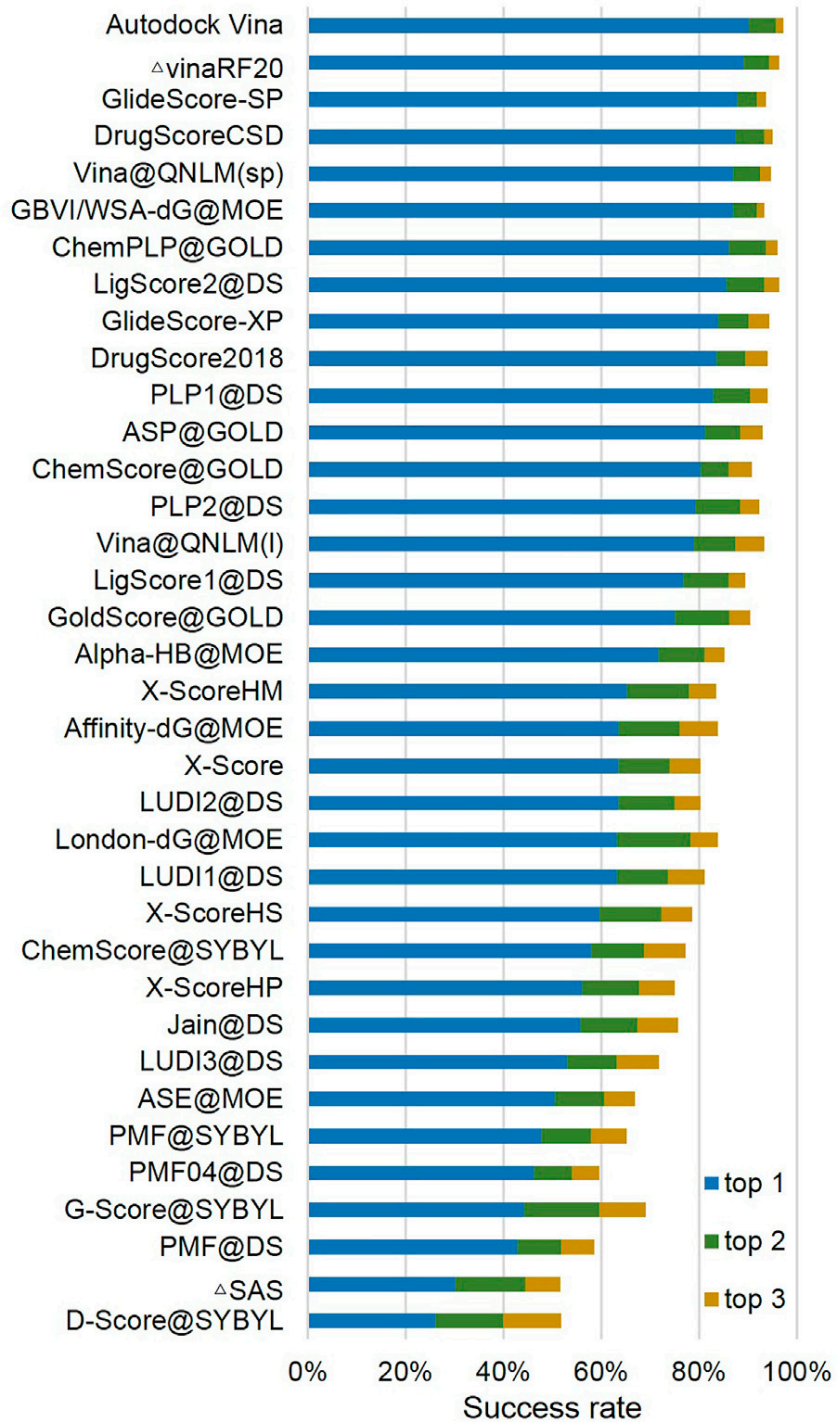
Docking power of top 1 (1%), top 2 (5%), and top 3 (10%) success rates of Vina@QNLM in single-point (sp) and local (l) modes (blue, green, and orange bars), compared to the methods evaluated in CASF-2016 and recalculated.

In the docking power test, each scoring function detected the success rate of the native ligand-binding site (RMSD <2.0 Å). Similar to the CASF-2013 dataset, we also divided the docking ability into three parts, namely, top 1 (1%), top 2 (5%), and top 3 (10%). AutoDock Vina showed superior performance in all the three sections. Vina@QNLM is in the middle of the top 1% but not far behind in the top 5%. It reached 93.3% in the local mode and 94.70% in the single-point mode (see Figure 9; Supplementary Table S10).

**FIGURE 10:**
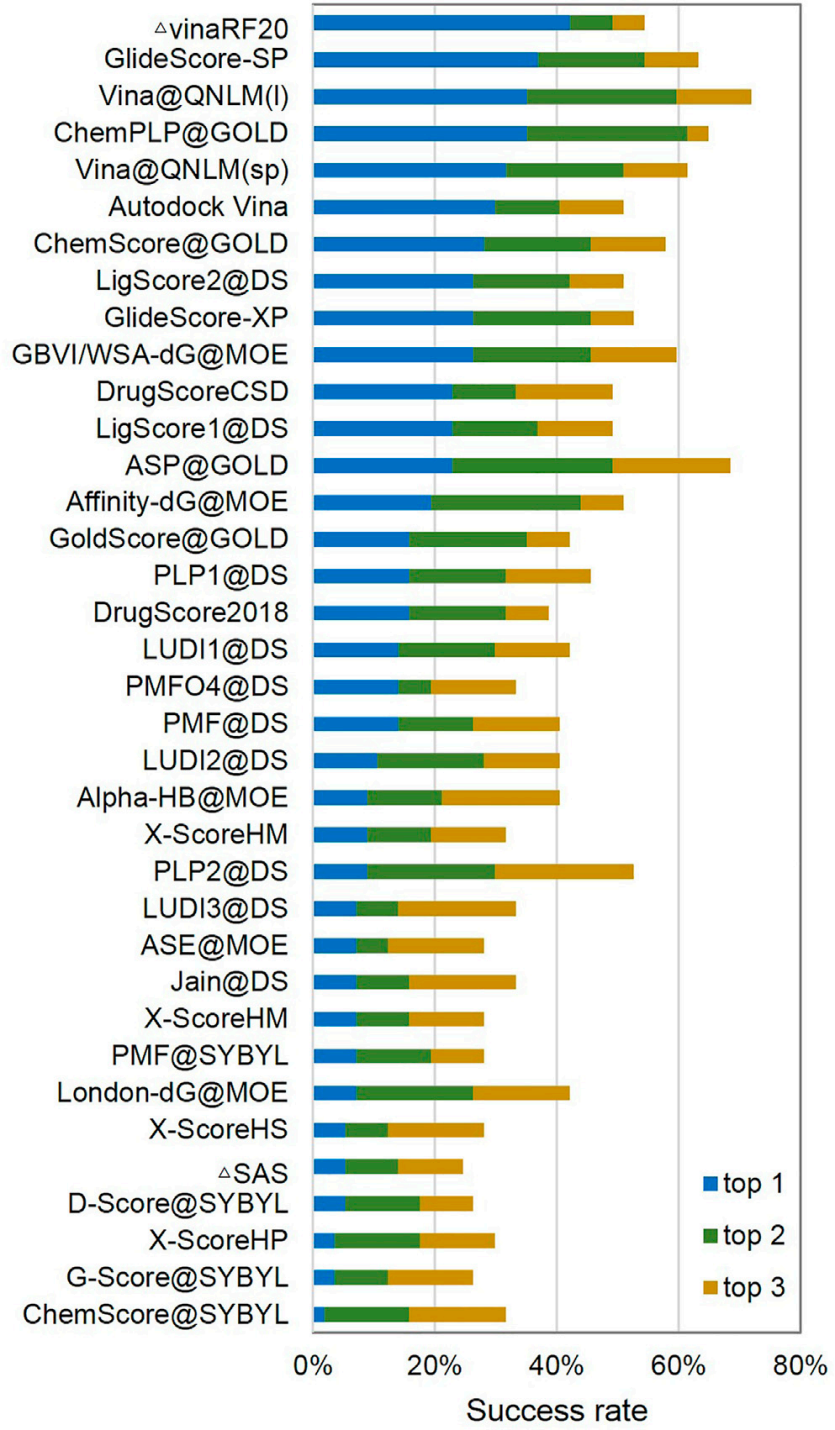
Screening power of top 1 (1%), top 2 (5%), and top 3 (10%) forward screening success rates of Vina@QNLM in single-point and local modes (blue, green, and orange bars), compared to the methods evaluated in CASF-2016 and recalculated.

In CASF-2016, the forward screening power of a scoring function is measured by its success rate of identifying the highest-affinity ligand for a given target protein. The success rates by considering the 1, 5, and 10% candidates in screening for all the scoring functions are illustrated in Figure 10. The data for making this figure are presented in Supplementary Table S11. In this test, the top five scoring functions, namely, VinaRF20, GlideScore-SP, ChemPLP@GOLD, Vina@QNLM(I), and Vina@QNLM(sp), have success rates above 30% at the top 1% level. Vina@QNLM(I) has the third highest success rate of 35.10%, only a little worse than those of VinaRF20 and GlideScore-SP. Considering the size of our test set, this scoring function has a chance of 35.10% to rank the highest-affinity ligand among the top three candidates.

**FIGURE 11:**
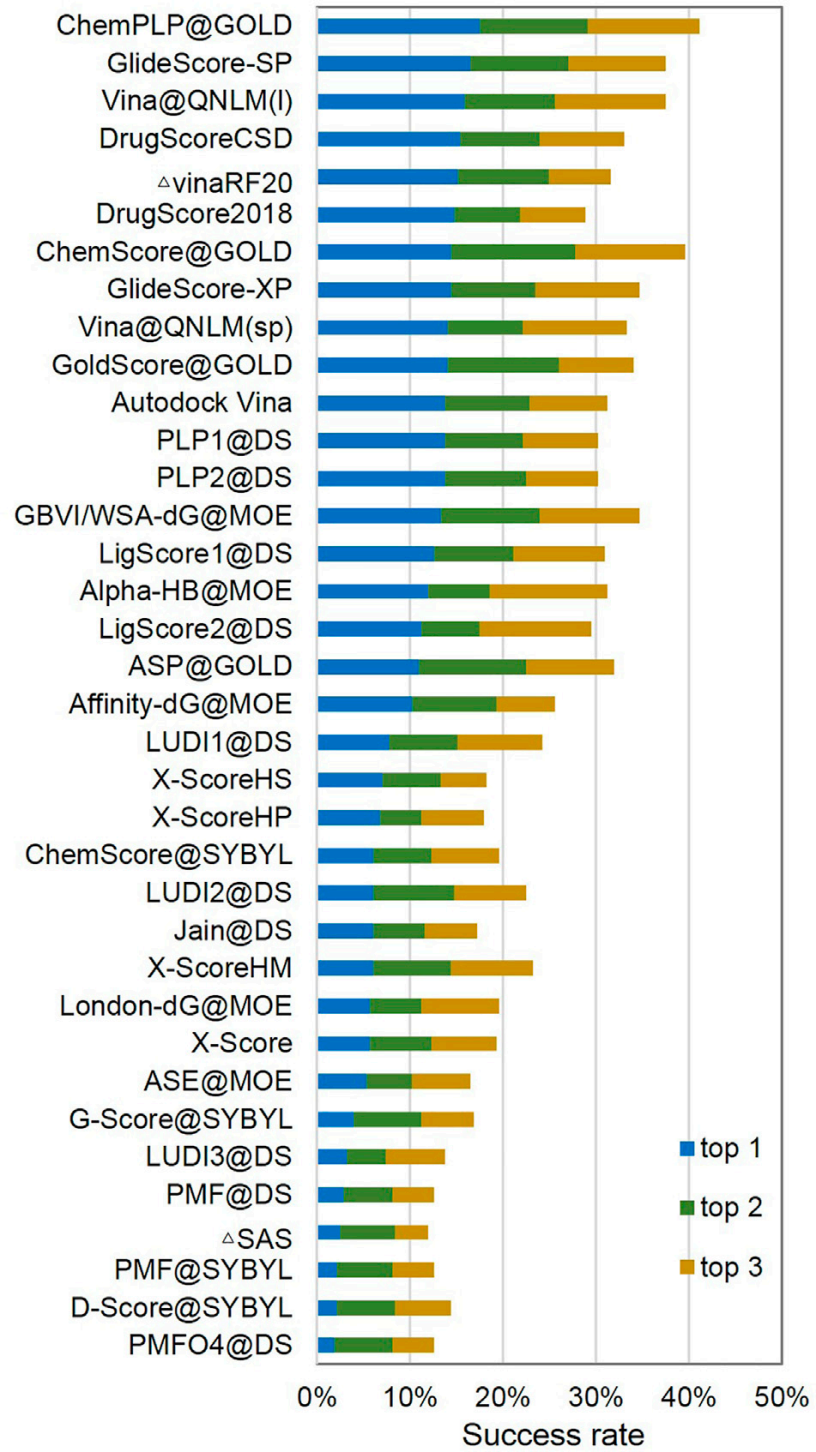
Screening power of top 1 (1%), top 2 (5%), and top 3 (10%) reverse screening success rates of Vina@QNLM in single-point and local modes (blue, green, and orange bars), compared to the methods evaluated in CASF-2016 and recalculated.

In CASF-2016, the screening power was also evaluated in a reverse screening trial. Here, the reverse screening power of a scoring function was measured by its success rate of identifying the true target protein for a given ligand molecule. The success rates by considering the top 1 (1%), top 5 (5%), and top 10 (10%) candidates in the screening for all the scoring functions are illustrated in Figure 11; Supplementary Table S12. In this test, the top five scoring functions are ChemPLP@GOLD, GlideScore-SP, Vina@QNLM(I), DrugScoreCSD, and VinaRF20. Their success rates of selecting the true target protein as the best-scored candidate are over 15%. Here, Vina@QNLM(l) has the third highest success rate of 15.80%, only a little worse than those of ChemPLP@GOLD and GlideScore-SP. These top-ranked scoring functions are also generally top-ranked in the forward screening trial. However, their success rates in the reverse screening trial are only half (or even lower) compared to their performance in the forward screening trial. For example, the success rates of Vina@QNLM(I) are 35.10 and 15.80% in the forward screening and reverse screening trials, respectively.

##### Sample Power

**FIGURE 12:**
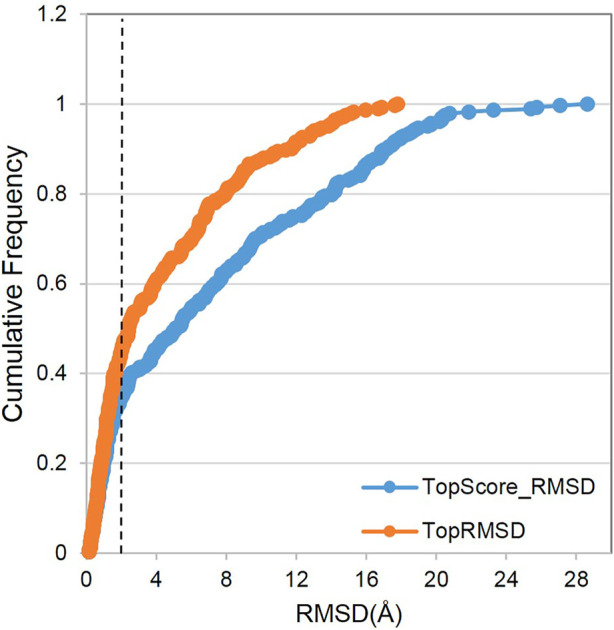
Sample power by comparing cumulative frequencies of TopScore_RMSD and TopRMSD, compared to the methods evaluated in CASF-2016 and recalculated.

In the evaluation of the conformation search ability of molecular docking, when the RMSD between the simulated value and experimental value is less than 2.0 Å, the simulation is considered to be effective, ranking fifth in the top magician RMSD mode and sixth in the best magician RMSD mode. The figure indicates that the success rate of Vina@QNLM for the top-scored pose is 38%, but that for the top RMSD pose is slightly higher (43%) (see Figure 12). By referring to other molecular docking software, the prediction ability of Vina@QNLM is at a medium level.

### Speedup Effect Comparison

The docking time is proportional to the number of active rotatable bonds in a ligand. Therefore, we used two sets of data to measure the acceleration effect, including the number of rotating bonds between 0–6 and 7–12. The elapsed time of molecular docking was measured by calculating the average time per receptor–ligand pair of a single molecule and a single target.

In this study, we used an Intel Core i7-4610 M processor to test the molecular docking times under the X86 platform. The docking time was positively correlated with the number of receptor–ligand pairs, with the figure increasing modestly from 1,976.35 s to approximately 19,378.34 s, and the receptor–ligand pairs were 100 and 800 (see Supplementary Table S13). Molecular docking with the number of rotating bonds between 7 and 12 showed the same tendency (see Supplementary Table S14).

**FIGURE 13:**
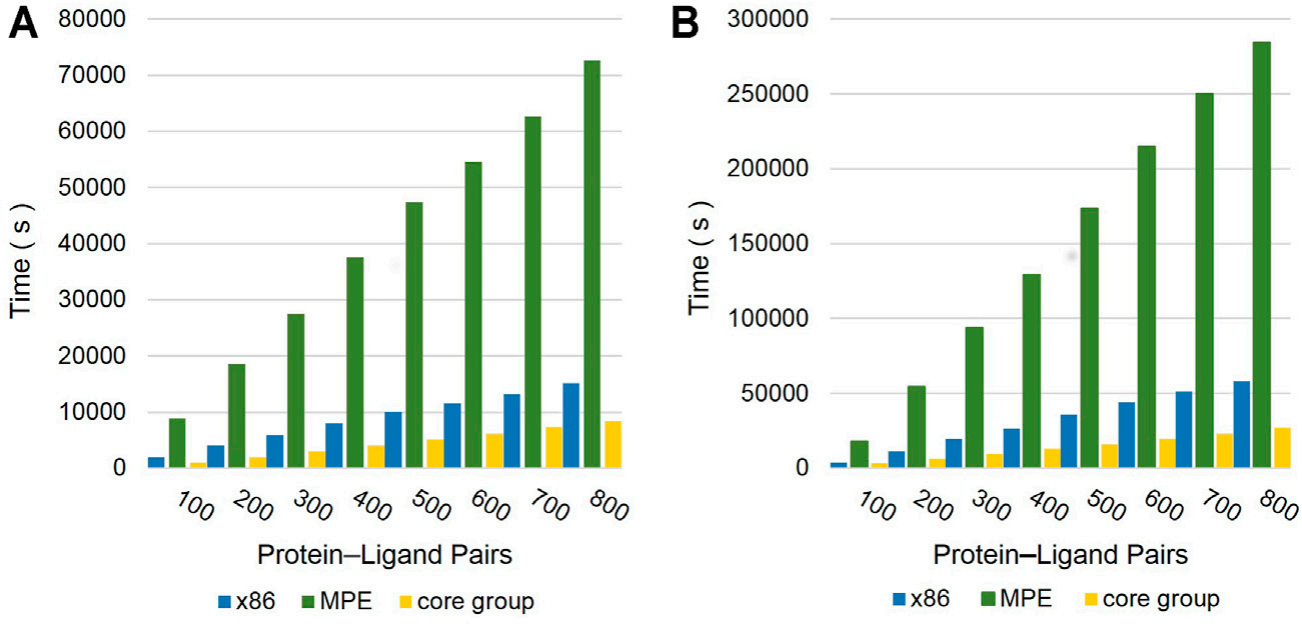
Comparison of the calculation speed in three ways: X86, MPE, and the core group. **(A)** The torsion of the docking molecule is 0–6. **(B)** The torsion of the docking molecule is 7–12.

The docking speed on Sunway’s MPE was significantly reduced as compared to the elapsed time of X86 owing to the hardware characteristics of the processor (see Figure 13A). Furthermore, we employed different compilation optimization methods provided by Sunway, but the computing time did not significantly improve. As a heterogeneous multicore processor, it is important to properly use the core to speed up the computation time. We completed the migration of the algorithm and guaranteed the maximum utilization of the CPE. By calculating the average docking time per receptor–ligand pair of a single molecule and single target, we can observe that the optimized Vina@QNLM elapsed time is faster than that of the X86 platform and previous version of Vina@QNLM (Table 1). For both ligands, the number of rotating bonds between 0–6 and 7–12 has a large speedup. Compared with the execution time of the serial version on MPE, speedups of approximately 10× on average and 11.0× at best were achieved.

**TABLE 1:** Parallel efficiency of the total elapsed time and docking elapsed time.

Protein–ligand pair	Process	Total elapsed time (s)	Docking elapsed time (s)	Parallel efficiency (docking) (%)	Parallel efficiency (total) (%)
9,59,000	4,80,001	240.66	117.26	55.92	53.68
9,59,000	2,40,501	441.75	250.86	52.17	58.37
9,59,000	1,61,001	467.68	303.00	64.52	82.35
9,59,000	80,501	929.45	495.65	78.88	82.87
9,59,000	41,001	1752.41	844.38	90.91	86.30
9,59,000	20,501	3,319.41	2015.56	76.17	91.12
9,59,000	10,501	5,905.00	2,997.23	100.00	100.00

### Parallel Efficiency

**FIGURE 14:**
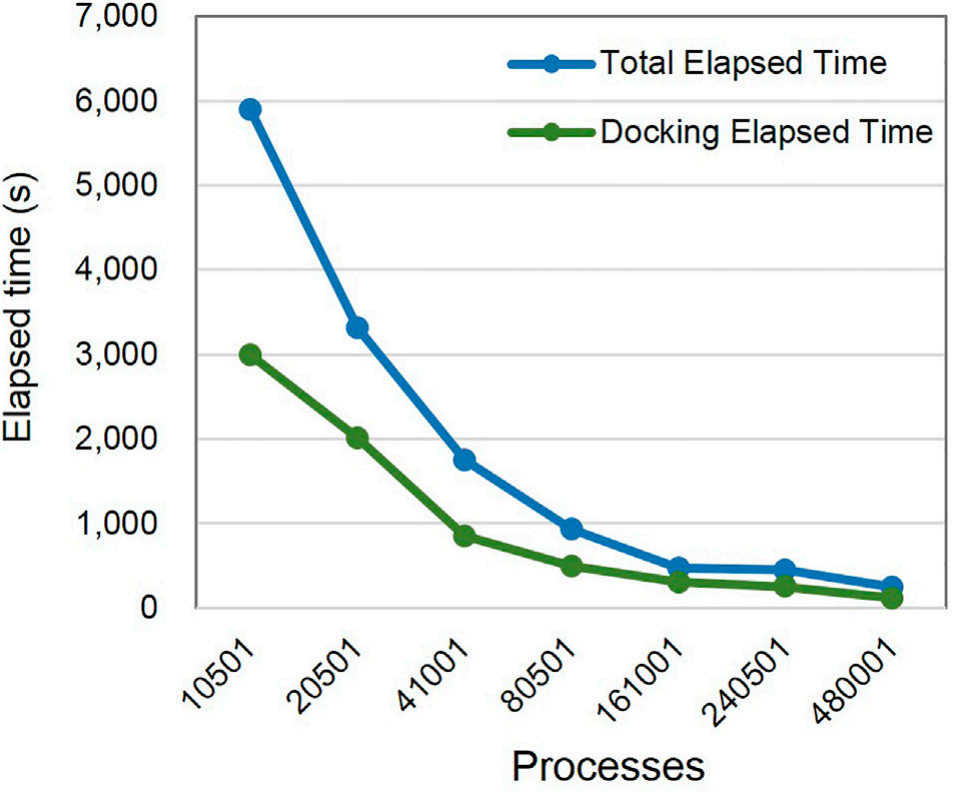
Scaling results on Sunway supercomputer.

As previously described, there are some barriers in the original molecular docking program to heterogeneous computers, such as the Sunway TaihuLight. Each process can be allocated a certain number of molecules to ensure a load balance based on MPI. Therefore, we employed two methods to evaluate the docking efficiency, that is, docking time and overall docking time, which included I/O and the time of sending molecular data. The computing time gradually decreased with the increase in the number of computing processes (see Figure 14).

We randomly selected 9,59,000 small molecules in the Zinc12 (Irwin et al., 2012) library, docked them with 6BFA protein, and recorded the elapsed time. The parallel efficiency of 10,501 processes was 100%, and the parallel efficiency slightly dropped with an increase in computing processes. A strong parallel efficiency of up to 55.92% was realized when Vina@QNLM was expanded to 4,80,001 processes. As for the parallel efficiency calculated based on the total elapsed time, the value was lower than the docking elapsed method, and 4,80,001 processes were used for docking, with a scalability of 53.68% (Table 1). Thus, Vina@QNLM can guarantee good scalability when run on Sunway supercomputer.

### Typical Applications

#### SARS-CoV-2

The global spread of the coronavirus disease continues to harm the public health. To screen molecules with promising medicinal purposes, first, we obtained 837,370,487 molecules in ZINC20 (http://zinc20.docking.org/tranches/home/). Then, almost 10 million (10,593,000) of these molecules were randomly selected. The molecules have a LogP between 1 and 4.5 and a molecular weight between 300 and 500. The 10 million molecules were docked with nine targets related to SARS-CoV-2: M^pro^, RdRp, RdRp_noMg, Spike-RBD, ACE2, NSP16, PL^pro^1, PL^pro^2, and X-domain**.** We used 161,001 processes (10,465,065 cores) for virtual screening and completed 95,337,000 times docking in 8.5 h. Some of the molecules were selected for further molecular dynamic experiments and clinical trials. The docking results for the top 1,000 compounds at each target can be seen in Supplementary Material result_Top1000.zip.

#### TRIP13 Target

Another typical application is the docking demand from the Shanghai Institute of Materia Medica of the Chinese Academy of Sciences. The Thyroid Hormone Receptor Interacting Protein 13 (TRIP13) target plays an important role in the preparation of tumor therapy drugs (Wang et al., 2019). Accordingly, Vina@QNLM is used to complete the docking of 100 million compounds with the TRIP13 target in 12 h on a parallel scale of 1,00,000 processes.

## Availability of Vina@QNLM

To ensure that the vast number of users can use our software, we developed an online system (http://vina.qnlm.ac/index.html). General users can submit docking tasks after registration and can use Vina@QNLM for molecular docking after being reviewed by the administrator.

## Conclusion

Molecular docking is important in the field of drug discovery. Large-scale and efficient molecular docking procedures can significantly shorten the lead compound discovery time. In this study, we propose an ultra-large molecular docking scheduling mechanism. We adopted the Vinardo software to fit the hardware architecture of the Sunway processor, which consisted of heterogeneous CGs (1 MPE and 64 CPEs). We realized molecular docking in the CPEs of Sunway’s CG through data exchange between the MPE and CPEs. The performance of Vina@QNLM was comprehensively evaluated using the CASF-2013 and CASF-2016 protein–ligand complex benchmarks and achieved an optimum of 11× speedup. We managed to expand to 10,465,065 cores, including 161,001 MPEs, reaching a strong scalability of 55.92%. Nine targets related to SARS-CoV-2 were docked with more than 10 million molecules, with a total of 95,337,000 times docking completed in 8.5 h. We also made the top 1,000 best-rated compounds available to the public for targets related to SARS-CoV-2 and developed a platform for ordinary users to submit docking tasks. Our ultra-large molecular docking scheduling mechanism and optimization methods can inspire other software adaptations in Sunway and other heterogeneous multicore supercomputers.

## Data Availability

The original contributions presented in the study are included in the article/Supplementary Material, and further inquiries can be directed to the corresponding author.
