# Supplementary material for: Redesigning Vina@QNLM for Ultra-Large-Scale Molecular Docking and Screening on a Sunway Supercomputer
Source: Front Chem. 2021 Oct 28;9:750325. doi: 10.3389/fchem.2021.750325 (PMC8581564; doi:10.3389/fchem.2021.750325)
Supplement: Supplementary file 2 [file DataSheet1.docx]

Supplementary Material

# Supplementary Data

## SARS-CoV-2

Docking results for the top 1000 compounds at each target can be seen in result_Top1000.zip.

# Supplementary Figures and Tables

## Supplementary Figures


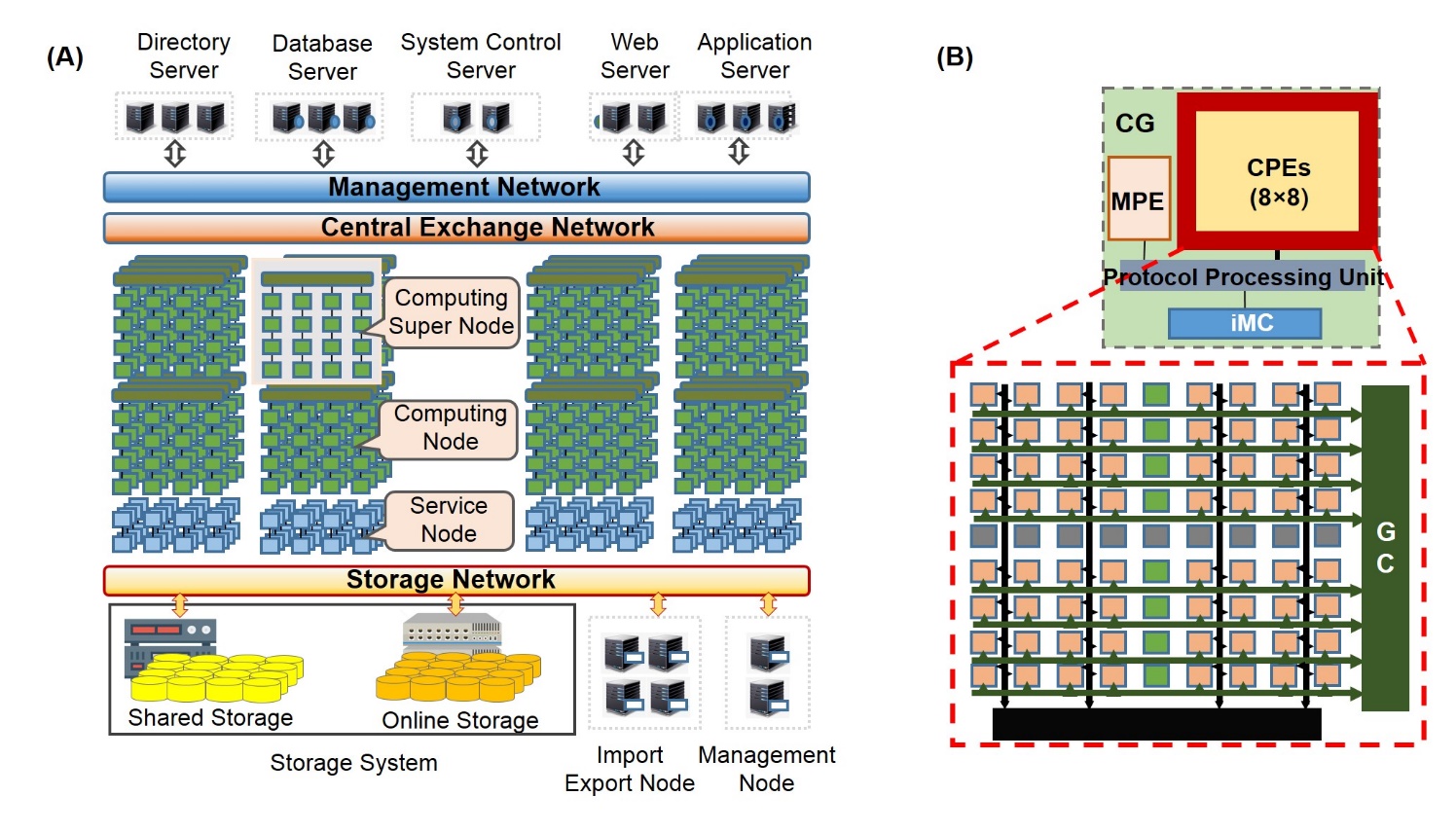


Supplementary Figure 1. Sunway supercomputing architecture (A) and Core group (B).

## Supplementary Tables

Supplementary Table S1. Scoring power of crystal structures and optimized structures pearson correlation coefficient of Vina@QNLM, compared to the methods evaluated in CASF-2013 and recalculated.

|  | crystal structures | | | optimized structures | | |
| --- | --- | --- | --- | --- | --- | --- |
| scoring function | N^b^ | R^c^ | SD^d^ | N | R | SD |
| X-Score^HM^ | 195 | 0.614 | 1.780 | 195 | 0.624 | 1.760 |
| ΔSAS | 195 | 0.606 | 1.790 | 195 | 0.596 | 1.810 |
| [ChemScore@SYBYL](mailto:ChemScore@SYBYL) | 195 | 0.592 | 1.820 | 194 | 0.546 | 1.890 |
| ChemPLP@GOLD | 195 | 0.579 | 1.840 | 195 | 0.538 | 1.900 |
| [PLP1@DS](mailto:PLP1@DS) | 195 | 0.568 | 1.860 | 195 | 0.557 | 1.870 |
| [G-Score@SYBYL](mailto:G-Score@SYBYL) | 195 | 0.558 | 1.870 | 189 | 0.456 | 2.010 |
| [ASP@GOLD](mailto:ASP@GOLD) | 195 | 0.556 | 1.880 | 195 | 0.529 | 1.920 |
| [ASE@MOE](mailto:ASE@MOE) | 195 | 0.544 | 1.890 | 195 | 0.547 | 1.890 |
| [ChemScore@GOLD](mailto:ChemScore@GOLD) | 189 | 0.536 | 1.900 | 187 | 0.473 | 1.990 |
| [D-Score@SYBYL](mailto:D-Score@SYBYL) | 195 | 0.526 | 1.920 | 194 | 0.530 | 1.910 |
| [Alpha-HB@MOE](mailto:Alpha-HB@MOE) | 195 | 0.511 | 1.940 | 193 | 0.487 | 1.970 |
| [LUDI3@DS](mailto:LUDI3@DS) | 195 | 0.487 | 1.970 | 195 | 0.448 | 2.020 |
| GoldScore@GOLD | 189 | 0.483 | 1.970 | 192 | 0.479 | 1.970 |
| [Affinity-dG@MOE](mailto:Affinity-dG@MOE) | 195 | 0.482 | 1.980 | 193 | 0.480 | 1.980 |
| [LigScore2@DS](mailto:LigScore2@DS) | 190 | 0.456 | 2.020 | 183 | 0.390 | 2.070 |
| GlideScore-SP | 169 | 0.452 | 2.030 | 155 | 0.425 | 2.110 |
| [Jain@DS](mailto:Jain@DS) | 191 | 0.408 | 2.050 | 194 | 0.341 | 2.130 |
| [PMF@DS](mailto:PMF@DS) | 194 | 0.364 | 2.110 | 193 | 0.357 | 2.100 |
| GlideScore-XP | 164 | 0.277 | 2.180 | 149 | 0.336 | 2.220 |
| [London-dG@MOE](mailto:London-dG@MOE) | 195 | 0.242 | 2.190 | 195 | 0.252 | 2.180 |
| [PMF@SYBYL](mailto:PMF@SYBYL) | 191 | 0.211 | 2.200 | 190 | 0.189 | 2.190 |
| AutoDock(single point) | 184 | 0.547 | 1.880 | 172 | 0.536 | 1.910 |
| AutoDock(local) | 194 | 0.592 | 1.820 | 195 | 0.581 | 1.840 |
| Vina(single point) | 195 | 0.564 | 1.860 | 191 | 0.536 | 1.900 |
| Vina(local) | 195 | 0.600 | 1.810 | 195 | 0.581 | 1.840 |
| [Vina@QNLM(single point)](mailto:Vina@QNLM(sp)) | 193 | 0.625 | 1.76 | 192 | 0.534 | 1.9 |
| [Vina@QNLM(local)](mailto:Vina@QNLM(l)) | 195 | 0.641 | 1.73 | 193 | 0.624 | 1.74 |

Supplementary Table S2. Ranking power of high and low-level success rate of Vina@QNLM, compared to the methods evaluated in CASF-2013 and recalculated.

|  | success rates(%) | | success rates(%) | |
| --- | --- | --- | --- | --- |
|  | on crystal structures | | on optimized structures | |
| scoring function | high-level^b^ | low-level^c^ | high-level | low-level |
| X-Score^HM^ | 58.500 | 72.300 | 56.900 | 73.800 |
| [ChemPLP@gold](mailto:ChemPLP@gold) | 58.500 | 72.300 | 46.200 | 61.500 |
| [PLP2@DS](mailto:PLP2@DS) | 55.400 | 72.300 | 47.700 | 67.700 |
| GoldScore@GOLD | 55.400 | 76.900 | 43.100 | 66.200 |
| ChemScore@SYBYL | 53.800 | 67.700 | 52.300 | 69.200 |
| Affinity-dG@MOE | 53.800 | 66.200 | 36.900 | 50.800 |
| LigScore2@DS | 52.300 | 61.500 | 50.800 | 63.100 |
| Alpha-HB@MOE | 52.300 | 66.200 | 47.700 | 64.600 |
| G-Score@SYBYL | 52.300 | 72.300 | 46.200 | 61.500 |
| [LUDI1@DS](mailto:LUDI1@DS) | 52.300 | 69.200 | 44.600 | 66.200 |
| D-Score@SYBYL | 49.200 | 63.100 | 52.300 | 63.100 |
| ΔSAS | 49.200 | 67.700 | 50.800 | 69.200 |
| PMF@DS | 49.200 | 66.200 | 46.200 | 63.100 |
| ASP@GOLD | 47.700 | 72.300 | 38.500 | 60.000 |
| ChemScore@GOLD | 46.200 | 63.100 | 33.800 | 53.800 |
| London-dG@MOE | 43.100 | 60.000 | 40.000 | 60.000 |
| PMF@SYBYL | 43.100 | 61.500 | 30.800 | 53.800 |
| GlideScore-SP | 43.100 | 56.900 | 21.500 | 38.500 |
| Jain@DS | 41.500 | 58.500 | 44.600 | 63.100 |
| ASE@MOE | 40.000 | 64.600 | 43.100 | 63.100 |
| GlideScore-XP | 35.400 | 47.700 | 32.300 | 46.200 |
| AutoDock(single point) | 50.800 | 70.800 | 41.500 | 53.800 |
| AutoDock(local) | 44.600 | 70.800 | 44.600 | 69.200 |
| Vina(single point) | 47.700 | 72.300 | 40.000 | 53.800 |
| Vina(local) | 49.200 | 69.200 | 44.600 | 67.700 |
| [Vina@QNLM(single point)](mailto:Vina@QNLM(sp)) | 18.500 | 33.800 | 29.200 | 46.200 |
| [Vina@QNLM(local)](mailto:Vina@QNLM(l)) | 55.400 | 73.800 | 38.500 | 56.900 |

Supplementary Table S3. Docking power (A) top 1 (1%), top 2 (5%), and top 3(10%) success rate of Vina@QNLM, compared to the methods evaluated in CASF-2013 and recalculated.

| scoring function | success rate(%) | | |
| --- | --- | --- | --- |
|  | top-1 ( % ) | top-2 ( % ) | top-3 ( % ) |
| ChemPLP@GOLD | 82 | 87 | 90 |
| ChemScore@GOLD | 81 | 85 | 89 |
| GlideScore-SP | 79 | 86 | 89 |
| Alpha HB@MOE | 75 | 85 | 89 |
| GoldScore@GOLD | 71 | 84 | 89 |
| ASP@GOLD | 72 | 82 | 88 |
| LigScore2@DS | 79 | 85 | 87 |
| GlideScore-XP | 75 | 83 | 87 |
| PLP1@DS | 78 | 84 | 86 |
| LUD12@DS | 68 | 79 | 85 |
| Affinity dG@MOE | 64 | 75 | 82 |
| London dG@MOE | 63 | 77 | 80 |
| ChemScore@SYBYL | 61 | 72 | 80 |
| X-Score(HM) | 63 | 75 | 79 |
| Gscore@SYBYL | 48 | 64 | 75 |
| Jain@DS | 51 | 65 | 73 |
| ASE@MOE | 53 | 63 | 67 |
| PMF@DS | 53 | 63 | 67 |
| PMF@SYBYL | 53 | 62 | 67 |
| dSAS | 27 | 38 | 49 |
| DScore@SYBYL | 21 | 33 | 47 |
| AutoDock(single point) | 74.4 | 84.1 | 85.6 |
| AutoDock(local) | 67.2 | 80.5 | 86.7 |
| Vina(single point) | 85.6 | 90.8 | 92.8 |
| Vina(local) | 77.4 | 85.1 | 89.2 |
| [Vina@QNLM (single point)](mailto:Vina@QNLM(sp)) | 61 | 75.9 | 86.2 |
| [Vina@QNLM(local)](mailto:Vina@QNLM(l)) | 70.8 | 81.5 | 84.6 |

Supplementary Table S4. Screening power top 1%, top 5% , and top 10% success rates (A) of Vina@QNLM, compared to the methods evaluated in CASF-2013 and recalculated.

| scoring function | success of finding the best ligand molecule among(%) | | |
| --- | --- | --- | --- |
|  | top 1% | top 5% | top 10% |
| GlideScore-SP | 60 | 72.3 | 76.9 |
| GlideScore-XP | 52.3 | 69.2 | 73.8 |
| ChemScore@GOLD | 49.2 | 78.5 | 83.1 |
| LigScore2@DS | 47.7 | 75.4 | 83.1 |
| [ChemPLP@GOLD](mailto:ChemPLP@GOLD) | 41.5 | 70.8 | 84.6 |
| [LUDI2@DS](mailto:LUDI2@DS) | 38.5 | 53.8 | 66.2 |
| ASP@GOLD | 36.9 | 75.4 | 81.5 |
| Affinity-dG@MOE | 23.1 | 50.8 | 66.2 |
| PLP1@DS | 21.5 | 52.3 | 70.8 |
| GoldScore@COLD | 21.5 | 52.3 | 66.2 |
| London-dG@MOE | 21.5 | 36.9 | 49.2 |
| Jain@DS | 16.9 | 29.2 | 40 |
| ChemScore@SYBYL | 15.4 | 33.8 | 50.8 |
| Alpha-HB@MOE | 13.8 | 36.9 | 63.1 |
| [PMF@SYBYL](mailto:PMF@SYBYL) | 13.8 | 23.1 | 38.5 |
| PMFO4@DS | 12.3 | 30.8 | 47.7 |
| ASE@MOE | 12.3 | 30.8 | 38.5 |
| X-Score^HM^ | 9.23 | 21.5 | 32.3 |
| D-Score@YBYL | 6.15 | 20 | 24.6 |
| G-Score@SYBYL | 4.62 | 16.9 | 30.8 |
| ΔSAS | 3.08 | 15.4 | 24.6 |
| AutoDock(single point) | 29.2 | 49.2 | 67.7 |
| AutoDock(local) | 40 | 61.5 | 70.8 |
| Vina(single) | 44.6 | 60 | 72.3 |
| Vina(local) | 47.7 | 60 | 64.6 |
| [Vina@QNLM(single point)](mailto:Vina@QNLM(sp)) | 61.5 | 81.5 | 87.7 |
| [Vina@QNLM(local)](mailto:Vina@QNLM(l)) | 61.5 | 76.9 | 83.1 |

Supplementary Table S5. Screening power top 1%, top 5%, and top 10% enrichment factors of Vina@QNLM, compared to the methods evaluated in CASF-2013 and recalculated.

| scoring function | enrichment factor | | |
| --- | --- | --- | --- |
|  | top 1% | top 5% | top 10% |
| GlideScore-SP | 19.540 | 6.270 | 4.140 |
| [ChemScore@GOLD](mailto:ChemScore@GOLD) | 18.900 | 6.830 | 4.080 |
| GlideScore-XP | 16.810 | 6.020 | 4.070 |
| LigScore2@DS | 15.900 | 6.230 | 3.510 |
| [ChemPLP@GOLD](mailto:ChemPLP@GOLD) | 14.280 | 5.880 | 4.310 |
| [LUDI1@DS](mailto:LUDI1@DS) | 12.530 | 4.280 | 2.800 |
| ASP@GOLD | 12.360 | 6.230 | 3.790 |
| Affinity-dG@MOE | 8.210 | 4.150 | 3.190 |
| [London-dG@MOE](mailto:London-dG@MOE) | 8.080 | 3.360 | 2.510 |
| GoldScore@COLD | 7.950 | 4.520 | 3.160 |
| PLP1@DS | 6.920 | 4.280 | 3.040 |
| Jain@DS | 5.900 | 2.510 | 1.800 |
| PMF@SYBYL | 5.380 | 2.210 | 1.900 |
| ChemScore@SYBYL | 5.260 | 2.380 | 2.180 |
| Alpha-HB@MOE | 4.870 | 3.230 | 1.320 |
| PMFO4@DS | 4.870 | 2.870 | 2.630 |
| ASE@MOE | 4.360 | 2.350 | 1.590 |
| X-Score^HM^ | 2.310 | 2.140 | 1.410 |
| D-Score@YBYL | 2.310 | 1.790 | 1.460 |
| G-Score@SYBYL | 1.920 | 1.260 | 1.440 |
| ΔSAS | 1.410 | 1.280 | 1.120 |
| AutoDock(single point) | 10.260 | 4.600 | 3.380 |
| AutoDock(local) | 15.010 | 5.480 | 3.550 |
| Vina(single point) | 15.570 | 5.550 | 3.670 |
| Vina(local) | 17.930 | 5.600 | 3.290 |
| Vina@QNLM(single point) | 19.540 | 6.650 | 4.020 |
| Vina@QNLM(local) | 20.110 | 6.720 | 4.050 |

Supplementary Table S6. Scoring power result obtained on the original complex structures, compared to the methods evaluated in CASF-2016 and recalculated.

| Scoring Function | Rank^a^ | N^b^ | R^c^ | SD^d^ |
| --- | --- | --- | --- | --- |
| △_vina_RF_20_ | 1 | 285 | 0.816 | 1.26 |
| [Vina@QNLM(singlepoint)](mailto:Vina@Qnlm(singlepoint)) | 2 | 285 | 0.641 | 1.67 |
| [Vina@QNLM(local)](mailto:Vina@Qnlm(local)) | 2 | 285 | 0.649 | 1.65 |
| X-Score | 3 | 285 | 0.631 | 1.69 |
| X-Score^HS^ | 4 | 285 | 0.629 | 1.69 |
| ASAS | 5 | 285 | 0.625 | 1.70 |
| X-Score^HP^ | 6 | 285 | 0.621 | 1.70 |
| [ASP@GOLD](mailto:ASP@GOLD) | 7 | 282 | 0.617 | 1.71 |
| ChemPLP@GOLD | 7 | 281 | 0.614 | 1.72 |
| X-Score^HM^ | 8 | 285 | 0.609 | 1.73 |
| Autodock Vina | 8 | 285 | 0.604 | 1.73 |
| DrugScore2018 | 8 | 285 | 0.602 | 1.74 |
| DrugScore^CSD^ | 9 | 285 | 0.596 | 1.75 |
| ASE@MOE | 10 | 285 | 0.591 | 1.75 |
| ChemScore@SYBYL | 10 | 285 | 0.59 | 1.76 |
| [PLP1@DS](mailto:PLP1@DS) | 11 | 285 | 0.581 | 1.77 |
| ChemScore@GOLD | 12 | 279 | 0.574 | 1.78 |
| G-Score@SYBYL | 13 | 284 | 0.572 | 1.79 |
| Alpha-HB@MOE | 14 | 285 | 0.569 | 1.79 |
| PLP2@DS | 15 | 285 | 0.563 | 1.80 |
| Affinity-dG@MOE | 16 | 285 | 0.552 | 1.81 |
| LigScore2@Ds | 17 | 285 | 0.54 | 1.83 |
| D-Score@SYBYL | 18 | 284 | 0.531 | 1.84 |
| LUDI2(@DS | 18 | 285 | 0.526 | 1.85 |
| GlideScore-SP | 19 | 258 | 0.513 | 1.89 |
| LUDI3@DS | 20 | 285 | 0.502 | 1.88 |
| GBVI/WSA-dG@MOE | 20 | 271 | 0.496 | 1.91 |
| LUDI1@DS | 21 | 284 | 0.494 | 1.88 |
| GlideScore-XP | 22 | 252 | 0.467 | 1.95 |
| Jain@DS | 23 | 279 | 0.457 | 1.89 |
| LigScore1@DS | 24 | 285 | 0.425 | 1.97 |
| PMF@DS | 24 | 282 | 0.422 | 1.97 |
| GoldScore@GOLD | 24 | 244 | 0.416 | 1.99 |
| London-dG@MOE | 25 | 285 | 0.405 | 1.99 |
| PMF@SYBYL | 26 | 279 | 0.262 | 2.09 |
| PMF04@DS | 27 | 263 | 0.212 | 2.11 |

Supplementary Table S7. Scoring power result obtained on the locally optimized complex structures, compared to the methods evaluated in CASF-2016 and recalculated.

| **Scoring Function** | **Rank^a^** | **N^b^** | **R^c^** | **SD^d^** |
| --- | --- | --- | --- | --- |
| △_vina_RF_20_ | 1 | 285 | 0.774 | 1.38 |
| [Vina@QNLM(single point)](mailto:Vina@Qnlm(singlepoint)) | 2 | 285 | 0.633 | 1.68 |
| [Vina@QNLM(local)](mailto:Vina@Qnlm(local)) | 2 | 285 | 0.642 | 1.67 |
| GBVI/WSA-dG@MOE | 3 | 277 | 0.633 | 1.69 |
| X-Score | 3 | 285 | 0.629 | 1.69 |
| X-Score^HS^ | 3 | 285 | 0.628 | 1.69 |
| △SAS | 4 | 285 | 0.621 | 1.70 |
| X-Score^HP^ | 5 | 285 | 0.62 | 1.71 |
| ASP@GOLD | 6 | 283 | 0.612 | 1.72 |
| X-Score^HM^ | 7 | 285 | 0.607 | 1.73 |
| ChemPLP@GOLD | 8 | 283 | 0.606 | 1.73 |
| DrugScore2018 | 9 | 285 | 0.596 | 1.75 |
| DrugScore^CSD^ | 10 | 285 | 0.593 | 1.75 |
| ASE@MOE | 10 | 285 | 0.592 | 1.75 |
| ChemScore@SYBYL | 11 | 285 | 0.584 | 1.76 |
| AIpha-HB@MOE | 12 | 285 | 0.578 | 1.77 |
| Autodock Vina | 13 | 285 | 0.578 | 1.77 |
| PLP1@DS | 14 | 285 | 0.573 | 1.78 |
| GlideScore-SP | 15 | 271 | 0.565 | 1.81 |
| ChemScore@GOLD | 16 | 276 | 0.562 | 1.80 |
| PLP2@DS | 17 | 285 | 0.559 | 1.80 |
| Affinity-dG@MOE | 18 | 285 | 0.553 | 1.81 |
| G-Score@SYBYL | 19 | 283 | 0.525 | 1.85 |
| D-Score@SYBYL | 20 | 284 | 0.52 | 1.86 |
| LUDI2@DS | 21 | 285 | 0.504 | 1.88 |
| LUDI3@DS | 22 | 285 | 0.497 | 1.89 |
| LigScore2@DS | 23 | 284 | 0.481 | 1.91 |
| GlideScore-XP | 23 | 266 | 0.478 | 1.93 |
| LUDI1@DS | 24 | 284 | 0.473 | 1.91 |
| GoldScore@GOLD | 24 | 263 | 0.466 | 1.91 |
| Jain@DS | 25 | 281 | 0.457 | 1.90 |
| PMF@DS | 26 | 283 | 0.422 | 1.97 |
| London-dG@MOE | 27 | 285 | 0.412 | 1.98 |
| LigScore1@DS | 28 | 284 | 0.393 | 2.00 |
| PMF@SYBYL | 29 | 278 | 0.265 | 2.09 |
| PMF04@DS | 30 | 264 | 0.204 | 2.11 |

Supplementary Table S8. Ranking power result obtained on the original complex structures, compared to the methods evaluated in CASF-2016 and recalculated.

| **Scoring Function** | **Rank^a^** | **^ρb^** | **τ^c^** | **PI^d^** |
| --- | --- | --- | --- | --- |
| △_vina_RF_20_ | 1 | 0.750 | 0.686 | 0.761 |
| ChemPLP@GOLD | 2 | 0.633 | 0.537 | 0.657 |
| DrugScore^CSD^ | 2 | 0.630 | 0.544 | 0.663 |
| LUDI2@DS | 3 | 0.629 | 0.543 | 0.657 |
| [Vina@QNLM(single point)](mailto:Vina@Qnlm(singlepoint)) | 4 | 0.616 | 0.526 | 0.651 |
| LUDI1@DS | 4 | 0.612 | 0.534 | 0.640 |
| LigScore2@DS | 5 | 0.608 | 0.521 | 0.620 |
| DrugScore2018 | 5 | 0.607 | 0.530 | 0.637 |
| [Vina@QNLM(local)](mailto:Vina@Qnlm(local)) | 5 | 0.616 | 0.533 | 0.651 |
| X-Score | 5 | 0.604 | 0.529 | 0.638 |
| X-Score^HM^ | 5,6 | 0.603 | 0.522 | 0.641 |
| Affinity-dG@MOE | 6 | 0.604 | 0.519 | 0.619 |
| LigScore1@DS | 7 | 0.599 | 0.523 | 0.606 |
| ChemScore@SYBYL | 7,8 | 0.593 | 0.530 | 0.617 |
| London-dG@MOE | 8,9 | 0.593 | 0.526 | 0.609 |
| G-Score@SYBYL | 9 | 0.591 | 0.526 | 0.609 |
| PLP2@DS | 9 | 0.589 | 0.512 | 0.617 |
| △SAS | 10 | 0.588 | 0.498 | 0.612 |
| PLP1@DS | 11 | 0.582 | 0.509 | 0.605 |
| D-Score@SYBYL | 12 | 0.577 | 0.516 | 0.598 |
| X-Score^HP^ | 13 | 0.573 | 0.508 | 0.607 |
| ASP@GOLD | 14 | 0.553 | 0.474 | 0.582 |
| X-Score^HS^ | 15 | 0.547 | 0.469 | 0.577 |
| PMF@DS | 15 | 0.537 | 0.470 | 0.559 |
| Alpha-HB@MOE | 16 | 0.535 | 0.477 | 0.558 |
| LUDI3@DS | 16,17 | 0.532 | 0.449 | 0.564 |
| Autodock Vina | 17 | 0.528 | 0.453 | 0.557 |
| ChemScore@GOLD | 17 | 0.526 | 0.460 | 0.558 |
| Jain@DS | 18 | 0.521 | 0.448 | 0.545 |
| GBVI/WSA-dG@MOE | 19 | 0.489 | 0.421 | 0.504 |
| PMF04@DS | 20 | 0.481 | 0.386 | 0.497 |
| PMF@SYBYL | 21 | 0.449 | 0.379 | 0.478 |
| ASE@MOE | 22 | 0.439 | 0.372 | 0.466 |
| GlideScore-SP | 23 | 0.419 | 0.374 | 0.425 |
| GoldScore@GOLD | 24 | 0.284 | 0.242 | 0.283 |
| GlideScore-XP | 24 | 0.257 | 0.227 | 0.255 |

Supplementary Table S9. Ranking power result obtained on the optimized complex structures, compared to the methods evaluated in CASF-2016 and recalculated.

| **Scoring Function** | **Rank^a^** | **^ρb^** | **τ^c^** | **PI^d^** |
| --- | --- | --- | --- | --- |
| △_vina_RF_20_ | 1 | 0.674 | 0.614 | 0.691 |
| ChemPLP@GOLD | 2 | 0.618 | 0.540 | 0.647 |
| [Vina@QNLM(local)](mailto:Vina@Qnlm(local)) | 2 | 0.612 | 0.523 | 0.646 |
| X-Score^HM^ | 3 | 0.611 | 0.536 | 0.642 |
| G-Score@SYBYL | 4 | 0.602 | 0.540 | 0.625 |
| DrugScore2018 | 5 | 0.596 | 0.505 | 0.633 |
| LUDI2@DS | 6 | 0.595 | 0.527 | 0.620 |
| X-Score | 6 | 0.595 | 0.523 | 0.625 |
| GBVI/WSA-dG@MOE | 7 | 0.591 | 0.509 | 0.602 |
| DrugScore^CSD^ | 8 | 0.591 | 0.505 | 0.626 |
| △SAS | 9 | 0.589 | 0.515 | 0.608 |
| [Vina@QNLM(singlepoint)](mailto:Vina@Qnlm(singlepoint)) | 10 | 0.584 | 0.512 | 0.612 |
| PLP1@DS | 10 | 0.584 | 0.505 | 0.614 |
| D-Score@SYBYL | 11 | 0.572 | 0.505 | 0.589 |
| London-dG@MOE | 12 | 0.567 | 0.502 | 0.580 |
| PLP2@DS | 12 | 0.567 | 0.505 | 0.595 |
| X-Score^HP^ | 12 | 0.566 | 0.509 | 0.596 |
| X-Score^HS^ | 13 | 0.560 | 0.489 | 0.590 |
| Affinity-dG@MOE | 14 | 0.560 | 0.484 | 0.574 |
| ASP@GOLD | 15 | 0.542 | 0.463 | 0.569 |
| ChemScore@SYBYL | 15 | 0.542 | 0.474 | 0.572 |
| Alpha-HB@MOE | 16 | 0.535 | 0.463 | 0.566 |
| LUDI3@DS | 17 | 0.532 | 0.444 | 0.568 |
| LUDI1@DS | 18 | 0.529 | 0.453 | 0.556 |
| Jain@DS | 19 | 0.514 | 0.453 | 0.547 |
| PMF@DS | 19 | 0.505 | 0.435 | 0.533 |
| LigScore2@DS | 20 | 0.482 | 0.406 | 0.506 |
| LigScore1@DS | 21 | 0.473 | 0.417 | 0.495 |
| Autodock Vina | 22 | 0.470 | 0.414 | 0.512 |
| ChemScore@GOLD | 22,23 | 0.467 | 0.414 | 0.499 |
| PMF04@DS | 23 | 0.465 | 0.372 | 0.477 |
| ASE@MOE | 24 | 0.435 | 0.365 | 0.459 |
| PMF@SYBYL | 25 | 0.411 | 0.333 | 0.439 |
| GlideScore-SP | 26 | 0.394 | 0.358 | 0.414 |
| GoldScore@GOLD | 27 | 0.365 | 0.323 | 0.381 |
| GlideScore-XP | 28 | 0.305 | 0.249 | 0.311 |

Supplementary Table S10. Docking power Success rates when the native ligand binding pose is included in each decoy set, compared to the methods evaluated in CASF-2016 and recalculated.

| **Scoring Function** | **Rank^a^** | **TOP1^b^** | **TOP2^c^** | **TOP3^d^** |
| --- | --- | --- | --- | --- |
| Autodock Vina | 1 | 90.20% | 95.80% | 97.20% |
| △_vina_RF_20_ | 2 | 89.10% | 94.40% | 96.50% |
| GlideScore-SP | 3 | 87.70% | 91.90% | 93.70% |
| DrugScore^CSD^ | 4 | 87.40% | 93.30% | 95.10% |
| [Vina@QNLM(singlepoint)](mailto:Vina@Qnlm(singlepoint)) | 5 | 87.00% | 92.60% | 94.70% |
| GBVI/WSA-dG@MOE | 5 | 87.00% | 91.90% | 93.30% |
| ChemPLP@GOLD | 6 | 86.00% | 93.70% | 96.10% |
| LigScore2@DS | 7 | 85.60% | 93.30% | 96.50% |
| GlideScore-XP | 8 | 83.90% | 90.20% | 94.40% |
| DrugScore2018 | 9 | 83.50% | 89.50% | 94.00% |
| PLP1@DS | 10 | 82.80% | 90.50% | 94.00% |
| ASP@GOLD | 11 | 81.10% | 88.40% | 93.00% |
| ChemScore@GOLD | 12 | 80.40% | 86.00% | 90.90% |
| PLP2@DS | 13 | 79.30% | 88.40% | 92.30% |
| [Vina@QNLM(local)](mailto:Vina@Qnlm(local)) | 14 | 78.90% | 87.40% | 93.30% |
| LigScore1@DS | 15 | 76.80% | 86.00% | 89.50% |
| GoldScore@GOLD | 16 | 75.10% | 86.30% | 90.50% |
| Alpha-HB@MOE | 17 | 71.60% | 81.10% | 85.30% |
| X-Score^HM^ | 18 | 65.30% | 77.90% | 83.50% |
| LUDI2@DS | 19 | 63.50% | 75.10% | 80.40% |
| X-Score | 19 | 63.50% | 74.00% | 80.40% |
| Affinity-dG@MOE | 19,20 | 63.50% | 76.10% | 83.90% |
| LUDI1@DS | 20 | 63.20% | 73.70% | 81.10% |
| London-dG@MOE | 20 | 63.20% | 78.20% | 83.90% |
| X-Score^HS^ | 21 | 59.60% | 72.30% | 78.60% |
| ChemScore@SYBYL | 22 | 57.90% | 68.80% | 77.20% |
| X-Score^HP^ | 23 | 56.10% | 67.70% | 75.10% |
| Jain@DS | 23 | 55.80% | 67.40% | 75.80% |
| LUDI3@DS | 24 | 53.00% | 63.20% | 71.90% |
| ASE@MOE | 25 | 50.50% | 60.70% | 67.00% |
| PMF@SYBYL | 26 | 47.70% | 57.90% | 65.30% |
| PMF04@DS | 27 | 46.30% | 54.00% | 59.60% |
| G-Score@SYBYL | 28 | 44.20% | 59.60% | 69.10% |
| PMF@DS | 29 | 42.80% | 51.90% | 58.60% |
| △SAS | 30 | 30.20% | 44.60% | 51.60% |
| D-Score@SYBYL | 31 | 26.00% | 40.00% | 51.90% |

Supplementary Table S11. screening power Success rates of identifying the best ligand for all scoring functions in the forward, compared to the methods evaluated in CASF-2016 and recalculated.

| **Scoring function** | **Rank^a^** | **Success rate(%)** | | |
| --- | --- | --- | --- | --- |
|  |  | Top1%^b^ | top5%^c^ | Top10%^d^ |
| △_vina_RF_20_ | 1 | 42.10% | 49.10% | 54.40% |
| GlideScore-SP | 2 | 36.80% | 54.40% | 63.20% |
| [Vina@QNLM(local)](mailto:Vina@Qnlm(local)) | 3 | 35.10% | 59.60% | 71.90% |
| ChemPLP@GOLD | 3 | 35.10% | 61.40% | 64.90% |
| [Vina@QNLM(singlepoint)](mailto:Vina@Qnlm(singlepoint)) | 4 | 31.60% | 50.90% | 61.40% |
| Autodock Vina | 5 | 29.80% | 40.40% | 50.90% |
| ChemScore@GOLD | 6 | 28.10% | 45.60% | 57.90% |
| GBVI/WSA-dG@MOE | 7 | 26.30% | 45.60% | 59.60% |
| GlideScore-XP | 7 | 26.30% | 45.60% | 52.60% |
| LigScore2@DS | 7 | 26.30% | 42.10% | 50.90% |
| ASP@GOLD | 8 | 22.80% | 49.10% | 68.40% |
| LigScore1@DS | 8 | 22.80% | 36.80% | 49.10% |
| DrugScore^CSD^ | 8 | 22.80% | 33.30% | 49.10% |
| Affinity-dG@MOE | 9 | 19.30% | 43.90% | 50.90% |
| DrugScore2018 | 10 | 15.80% | 31.60% | 38.60% |
| PLP1@DS | 10 | 15.80% | 31.60% | 45.60% |
| GoldScore@GOLD | 10 | 15.80% | 35.10% | 42.10% |
| PMF@DS | 11 | 14.00% | 26.30% | 40.40% |
| PMFO4@DS | 11 | 14.00% | 19.30% | 33.30% |
| LUDI1@DS | 11 | 14.00% | 29.80% | 42.10% |
| LUDI2@DS | 12 | 10.50% | 28.10% | 40.40% |
| PLP2@DS | 13 | 8.80% | 29.80% | 52.60% |
| X-Score^HM^ | 13 | 8.80% | 19.30% | 31.60% |
| Alpha-HB@MOE | 13 | 8.80% | 21.10% | 40.40% |
| London-dG@MOE | 14 | 7.00% | 26.30% | 42.10% |
| [PMF@SYBYL](mailto:PMF@SYBYL) | 14 | 7.00% | 19.30% | 28.10% |
| X-Score^HM^ | 14 | 7.00% | 15.80% | 28.10% |
| Jain@DS | 14 | 7.00% | 15.80% | 33.30% |
| [ASE@MOE](mailto:ASE@MOE) | 14 | 7.00% | 12.30% | 28.10% |
| LUDI3@DS | 14 | 7.00% | 14.00% | 33.30% |
| D-Score@SYBYL | 15 | 5.30% | 17.50% | 26.30% |
| △SAS | 15 | 5.30% | 14.00% | 24.60% |
| X-Score^HS^ | 16 | 5.30% | 12.30% | 28.10% |
| [G-Score@SYBYL](mailto:G-Score@SYBYL) | 17 | 3.50% | 12.30% | 26.30% |
| X-Score^HP^ | 18 | 3.50% | 17.50% | 29.80% |
| [ChemScore@SYBYL](mailto:ChemScore@SYBYL) | 19 | 1.80% | 15.80% | 31.60% |

Supplementary Table S12. Success rates of identifying the best target protein for all scoring functions in the reverse screening power test, compared to the methods evaluated in CASF-2016 and recalculated.

| **Scoring function** | **Rank^a^** | **Success rate (%)** | | |
| --- | --- | --- | --- | --- |
|  |  | Top1%^b^ | top5%^c^ | Top10%^d^ |
| ChemPLP@GOLD | 1 | 17.50% | 29.10% | 41.10% |
| GlideScore-SP | 2 | 16.50% | 27.00% | 37.50% |
| [Vina@QNLM(local)](mailto:Vina@Qnlm(local)) | 3 | 15.80% | 25.60% | 37.50% |
| DrugScore^CSD^ | 4 | 15.40% | 23.90% | 33.00% |
| △vinaRF_20_ | 5 | 15.10% | 24.90% | 31.60% |
| DrugScore2018 | 6 | 14.70% | 21.80% | 28.80% |
| GlideScore-XP | 7 | 14.40% | 23.50% | 34.70% |
| ChemScore@GOLD | 7 | 14.40% | 27.70% | 39.60% |
| [Vina@QNLM(singlepoint)](mailto:Vina@Qnlm(singlepoint)) | 8 | 14.00% | 22.10% | 33.30% |
| GoldScore@GOLD | 8 | 14.00% | 26.00% | 34.00% |
| PLP2@DS | 9 | 13.70% | 22.50% | 30.20% |
| PLP1@DS | 9 | 13.70% | 22.10% | 30.20% |
| Autodock Vina | 9 | 13.70% | 22.80% | 31.20% |
| GBVI/WSA-dG@MOE | 10 | 13.30% | 23.90% | 34.70% |
| LigScore1@DS | 11 | 12.60% | 21.10% | 30.90% |
| Alpha-HB@MOE | 12 | 11.90% | 18.60% | 31.20% |
| LigScore2@DS | 13 | 11.20% | 17.50% | 29.50% |
| ASP@GOLD | 14 | 10.90% | 22.50% | 31.90% |
| Affinity-dG@MOE | 15 | 10.20% | 19.30% | 25.60% |
| LUDI1@DS | 16 | 7.70% | 15.10% | 24.20% |
| X-Score^HS^ | 17 | 7.00% | 13.30% | 18.20% |
| X-Score^HP^ | 18 | 6.70% | 11.20% | 17.90% |
| X-Score^HM^ | 19 | 6.00% | 14.40% | 23.20% |
| Jain@DS | 19 | 6.00% | 11.60% | 17.20% |
| LUDI2@DS | 19 | 6.00% | 14.70% | 22.50% |
| ChemScore@SYBYL | 19 | 6.00% | 12.30% | 19.60% |
| X-Score | 20 | 5.60% | 12.30% | 19.30% |
| London-dG@MOE | 20 | 5.60% | 11.20% | 19.60% |
| ASE@MOE | 21 | 5.30% | 10.20% | 16.50% |
| G-Score@SYBYL | 22 | 3.90% | 11.20% | 16.80% |
| LUDI3@DS | 23 | 3.20% | 7.40% | 13.70% |
| PMF@DS | 24 | 2.80% | 8.10% | 12.60% |
| △SAS | 25 | 2.50% | 8.40% | 11.90% |
| D-Score@SYBYL | 26 | 2.10% | 8.40% | 14.40% |
| PMF@SYBYL | 26 | 2.10% | 8.10% | 12.60% |
| PMFO4@DS | 27 | 1.80% | 8.10% | 12.60% |

Supplementary Table S13. Molecular docking with the number of rotating bonds for 0–6 speedup on X86, MPE, and CG.

| **Receptor** | **Number of Ligands** | **X86 ( s )** | **MPE ( s )** | **CG ( s )** | **Speedup (CG : X86)** | **Speedup (CG : MPE)** | |
| --- | --- | --- | --- | --- | --- | --- | --- |
| 6BFA | 100 | 1,976.35 | 8830.42 | 951.709 | 2.08 | | 9.28 |
|  | 200 | 4,066.56 | 18572.52 | 1,981.73 | 2.05 | | 9.37 |
|  | 300 | 5,934.12 | 27529.70 | 2,973.56 | 2.00 | | 9.26 |
|  | 400 | 8,017.57 | 37612.29 | 3,993.98 | 2.01 | | 9.42 |
|  | 500 | 10,037.71 | 47399.76 | 5,090.73 | 1.97 | | 9.31 |
|  | 600 | 11,486.61 | 54511.74 | 6,154.6 | 1.87 | | 8.86 |
|  | 700 | 13,156.62 | 62617.78 | 7,309.51 | 1.80 | | 8.57 |
|  | 800 | 15,217.21 | 72649.57 | 8,405.38 | 1.81 | | 8.64 |

Supplementary Table 14 Molecular docking with the number of rotating bonds for 7–12 speedup on X86, MPE, and CG.

| **Receptor** | **Number of Ligands** | **X86 ( s )** | **MPE ( s )** | **CG ( s )** | **Speedup (CG : X86)** | **Speedup (CG : MPE)** |
| --- | --- | --- | --- | --- | --- | --- |
| 6BFA | 100 | 3,642.49 | 17,780.51 | 3,073.53 | 1.19 | 5.79 |
|  | 200 | 11,127.08 | 54,414.90 | 6,139.13 | 1.81 | 8.86 |
|  | 300 | 19,330.66 | 94,202.72 | 9,451.16 | 2.05 | 9.97 |
|  | 400 | 26,562.57 | 129,322.63 | 12,779.1 | 2.08 | 10.12 |
|  | 500 | 35,731.87 | 173,619.08 | 16,055.7 | 2.23 | 10.81 |
|  | 600 | 44,209.61 | 214,735.92 | 19,511.1 | 2.27 | 11.01 |
|  | 700 | 51,373.09 | 249,993.80 | 23,085.4 | 2.23 | 10.83 |
|  | 800 | 58,409.9 | 284,543.64 | 26,797.9 | 2.18 | 10.62 |
